# Identification of the RSX interactome in a marsupial shows functional coherence with the *Xist* interactome during X inactivation

**DOI:** 10.1186/s13059-024-03280-0

**Published:** 2024-05-23

**Authors:** Kim L. McIntyre, Shafagh A. Waters, Ling Zhong, Gene Hart-Smith, Mark Raftery, Zahra A. Chew, Hardip R. Patel, Jennifer A. Marshall Graves, Paul D. Waters

**Affiliations:** 1 School of Biotechnology and Biomolecular Sciences, University of New South Wales, Sydney, NSW 2052 Australia; 2 School of Biomedical Sciences, Faculty of Medicine and Health, University of New South Wales, Sydney, NSW 2052 Australia; 3 Bioanalytical Mass Spectrometry Facility, University of New South Wales, Sydney, NSW 2052 Australia; 4 Australian Proteome Analysis Facility, Macquarie University, Macquarie Park, NSW Australia; 5 National Centre for Indigenous Genomics, Australian National University, Canberra, ACT 2601 Australia; 6 Department of Environment and Genetics, La Trobe University, Melbourne, VIC 3086 Australia

**Keywords:** X chromosome inactivation, Convergent evolution, *RSX* protein interactome

## Abstract

The marsupial specific *RSX* lncRNA is the functional analogue of the eutherian specific *XIST*, which coordinates X chromosome inactivation. We characterized the *RSX* interactome in a marsupial representative (the opossum *Monodelphis domestica*), identifying 135 proteins, of which 54 had orthologues in the *XIST* interactome. Both interactomes were enriched for biological pathways related to RNA processing, regulation of translation, and epigenetic transcriptional silencing. This represents a remarkable example showcasing the functional coherence of independently evolved lncRNAs in distantly related mammalian lineages.

**Supplementary Information:**

The online version contains supplementary material available at 10.1186/s13059-024-03280-0.

## Background

The sex chromosomes of therian mammals (marsupials and eutherians) share a common ancestry [1], having evolved from a pair of autosomes [2] after the divergence of therian and monotreme mammals approximately 187 mya [3]. X chromosome inactivation (XCI) occurs in both groups of mammals, implying an ancient origin. However, XCI in eutherians and marsupials involve molecular mechanisms that are remarkably different.

XCI in therian mammals silences transcription of one of the two X chromosomes in female somatic cells [4]. It is established in the early embryo and maintained through subsequent cell divisions and serves as an important model for epigenetic silencing due to its unparalleled scale and stability. Long-noncoding RNAs (lncRNAs) have emerged as common regulators in therian XCI. Eutherian XCI is mediated by a lncRNA called *XIST* [5]. Its mouse orthologue, *Xist* [6], shares ~ 67% sequence conservation with human *XIST.* This includes a series of tandem repeats (A to F), of which only repeat A is well conserved across all eutheria [7].

The protein interactome of *Xist* has been investigated in mouse cell lines using techniques involving chromatin isolation by RNA precipitation with mass spectrometry (ChIRP-MS) and its variations [8–12]. These investigations have identified 494 proteins in total, with only 6 proteins (Hnrnpm, Hnrnpu, Myef2, Raly, RBM15, Spen) common to all studies [8–10, 12]. An alternative technique, RNA immunoprecipitation (RIP) combined with deep sequencing, identified epigenetic regulators in the human *XIST* interactome that were not identified in the mouse studies: EZH2 and SUZ12, subunits of polycomb repressive complex 2 (PRC2), and CHD4, a subunit of the NuRD histone deacetylase complex [13, 14].

Marsupials lack an *XIST* gene; instead, ancient protein-coding genes have been retained at the loci homologous to those from which *XIST* and neighbouring genes evolved in eutherians [15–17]. In marsupials, XCI is mediated by a lncRNA called *RSX* [18] that is derived from a non-homologous and physically distinct region of the X chromosome. *RSX* is 27 kb in *Monodelphis domestica* (grey short-tailed opossum) [18] and 30 kb in koala (*Phascolarctus cinereus*) [19], longer than the 15 kb mouse *Xist* [6] and the 17 kb human *XIST* [20]. Although lacking linear sequence homology, a k-mer analysis classified two major groupings of repeat domains that are shared between *Xist* and *RSX* (*RSX* repeat 1 with *Xist* repeats B, C and *XIST* repeat D, and *RSX* repeats 2, 3 and 4 with *Xist* repeats A and E). Each of these domains is enriched for specific protein binding motifs [21]. Therefore, although *RSX* and *Xist* share no sequence homology they could be functional analogues.

*Xist* and *RSX* are both nuclear transcripts that are spliced, capped and polyadenylated in the manner of mRNAs, and are expressed only in female somatic cells, exclusively from the inactive X chromosome. In both cases, the clustered transcripts can be visualised using RNA fluorescence in-situ hybridisation (RNA FISH) as a distinctive cloud-like signal accumulated on the inactive X chromosome [18, 20]. Induction of *RSX* expression from an autosomal transgene in mouse silences transcription *in cis* [18]. This indicates a silencing capacity similar to that of *Xist* [22], although marsupial XCI is ‘leakier’ or more incomplete than the *XIST*-driven process in eutherians [23], perhaps due to the evolution of two different lncRNAs in different ancestral genomic contexts.

Here, we investigate the protein interactome of *RSX* in a marsupial, *Monodelphis*, and compare it with the *Xist* protein interactome. We consider the molecular mechanisms underlying the convergent evolution of XCI in therian mammals to enhance understanding of the evolution of the adaptations for balancing gene expression between the sexes. Our findings show that *RSX* interactors significantly overlap with *Xist* interactors, falling within the same protein–protein association network related to RNA splicing and processing, translation regulation and ribosome biogenesis, and epigenetic transcriptional silencing. This highlights the remarkable functional coherence of these non-homologous and independently evolved lncRNAs. We identified overlap between the *Xist* and *RSX* protein interactomes, both of which are enriched for functions associated with post-transcriptional regulation of gene expression. Post-transcriptional regulation has been shown to contribute to the balancing of expression of X-borne genes between the sexes in eutherians [24–27], although the underlying mechanisms are unknown.

## Results

### Identification and validation of *RSX* interactors and comparison with *Xist* interactome

**Fig. 1:**
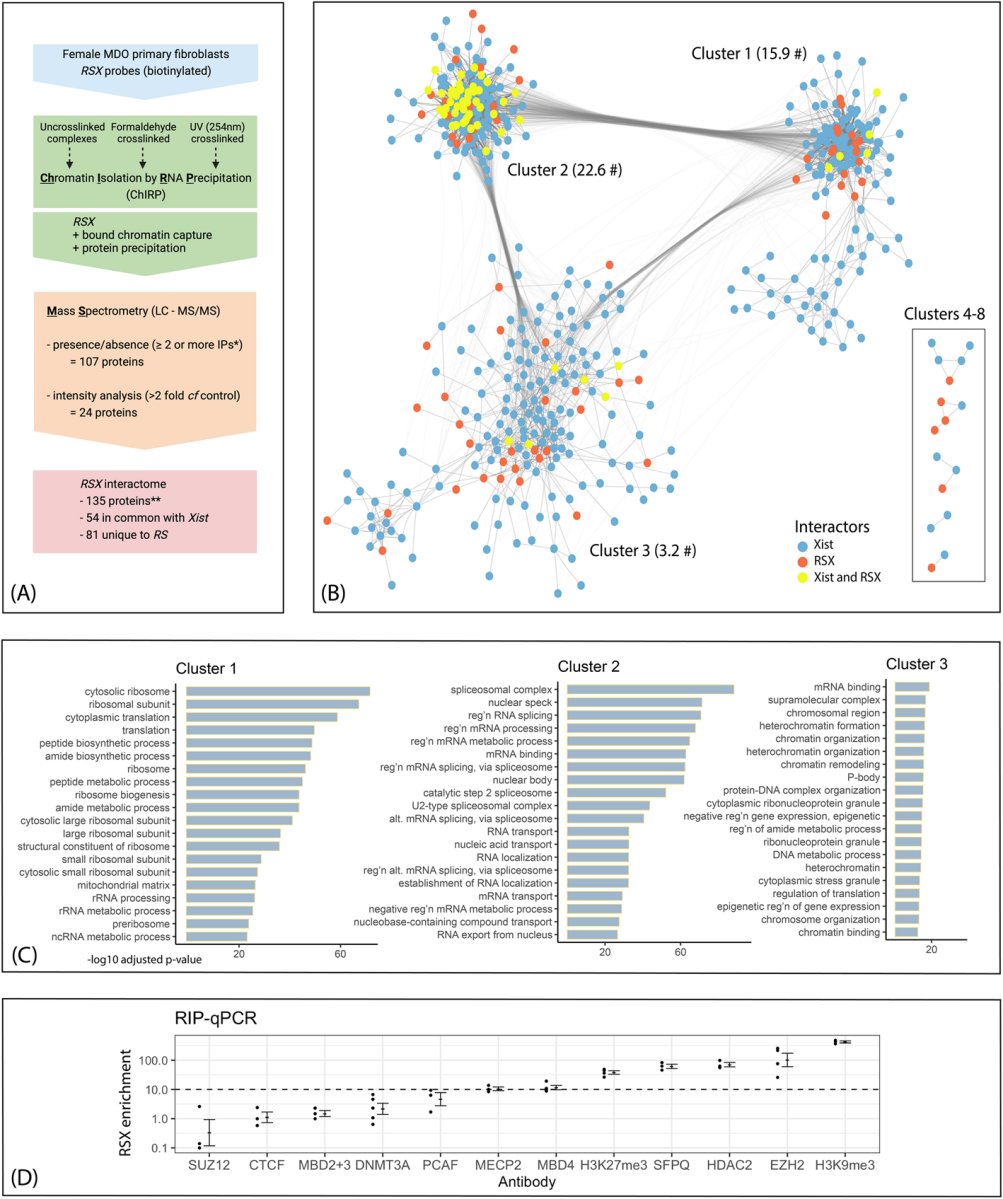
*RSX* and *Xist* interactomes share common orthologous proteins and protein–protein association networks with distinctive functional enrichments. **A** Overview of ChIRP-MS workflow. *Two proteins were identified by a single pulldown from a UV crosslinked sample. **includes 4 additional proteins identified using RIP-qPCR. Graphic created using BioRender.com. **B** Protein–protein interactions of the *RSX* and *Xist* interactomes based on experimentally determined interactions, co-expression, and curated database annotations for human orthologs (STRING database v11.5) [28]. Each node represents an interactome protein, each edge represents an annotated protein–protein interaction of minimum confidence 0.4. Interaction networks were visualised using Cytoscape (v3.8.2) [29], omitting proteins with no annotated interactions. Nodes were clustered based on connectivity (number and weight of edges) using the GLay Cytoscape plugin [30] with default settings. Intercluster edges to minor clusters (4–10) omitted for clarity. **#** denotes mean intracluster node degree (21). **C** Key functional and structural enrichments of each major protein interaction cluster. GSEA was conducted using gProfiler2 [31] with multiple testing correction based on false discovery rate. **D** Enrichment of *RSX* (fold change relative to Igg controls) by immunoprecipitation of protein targets from female *Monodelphis* fibroblast cell lysates, followed by quantitative PCR using *RSX*-specific primers. Enrichment (30-fold) was also detected for HNRNPK, as previously published [21]

To investigate the protein interactome of *RSX* we used ChIRP-MS to capture proteins associated with *RSX* using six biotinylated oligonucleotides complementary to different *RSX* regions (Additional file 1). Cell lysates were prepared from female *Monodelphis* fibroblast cells that were either UV crosslinked, formaldehyde crosslinked, or uncrosslinked. We identified 131 proteins that were associated with *RSX* using alternate criteria of presence/absence and greater than two-fold enrichment versus a control, either absence of oligonucleotides or scrambled oligonucleotides (Fig. 1A, Additional file 2).

We validated two *RSX* interactors using RNA immunoprecipitation (RIP) followed by quantitative PCR (qPCR). The RIP targets were chosen for their potential role in XCI. In eutherian models, SUZ12/EZH2 (core components of PRC2), HDAC2, HNRNPK and CTCF have roles in eutherian XCI. MBD2 + 3, MBD4, and MECP2 bind methylated DNA and are involved in chromatin remodelling and gene regulation. The histone marks H3K9me3 and H3K27me3 are known to accumulate on the inactive X in marsupials [32, 33]. PCAF is an acetyltransferase, so served as a negative control (Fig. 1D). Target proteins were immunoprecipitated from female *Monodelphis* fibroblast cell lysates, followed by qPCR using *RSX*-specific primers [21]. Enrichment of *RSX* (relative to IgG controls) of greater than tenfold was detected for seven targets. These included HNRNPK [21] and SFPQ, which were identified by the ChIRP-MS. The RIP-qPCR also identified four additional *RSX* interactors not detected by ChIRP-MS: EZH2 (PRC2 catalytic subunit), HDAC2 (a histone deacetylase), MBD4 (a methyl-CpG binding domain protein), and MECP2 (a methyl-CpG-binding protein).

These proteins were included in the *RSX* interactome, bringing the total to 135 proteins. We considered whether proteins of the *RSX* interactome had orthologues in the *Xist* interactome. Of the 135 *RSX*-interactors, 81 did not have orthologues in the *Xist* interactome, so were specific to the *RSX* interactome. The remaining 54 *RSX*-interactors had orthologues that were identified in the *Xist* interactome (which comprises 497 proteins in total). Therefore, we identified a substantial cohort of proteins that interact with both *RSX* and *Xist*, despite the lack of homology in the primary sequence of these two lncRNAs. We also considered the extent to which the two interactomes might include different proteins from common functional pathways, potentially providing insights into how therian XCI evolved to be mediated by different lncRNAs in marsupials and eutherians.

### Network analysis of the *RSX* and *Xist* interactomes reveals functional similarities

Gene set enrichment analysis (GSEA) [34] of each of the *RSX* and *Xist* interactomes identified that over 90% of the 136 ontology terms enriched for the *RSX* interactome were also enriched for the *Xist* interactome (*p* < 1 × 10^−3^) (Additional file 3: Tab 5), suggesting functional similarities between the two interactomes.

We queried the protein–protein interactions within the combined *RSX* and *Xist* interactomes using the STRING database (v11.5), with experimental findings, co-expression data, and evidence from curated databases as interaction sources [28]. Of the 578 proteins in the combined interactomes, 516 proteins had at least one interaction (confidence score > 0.4) and formed a network with 8721 edges (a mean of 15.1 edges per node). This was significantly higher than the 3633 edges expected for a random set of 578 proteins selected from the same proteome (*p* < 1 × 10^−16^). Clustering of the interaction network partitioned it into three larger clusters and five small clusters (Fig. 1B, Additional file 4: Tab 1). The key functional enrichments of each of the three major clusters were determined using GSEA. The three large clusters were individually enriched for functions including mRNA binding, translation (and regulation of translation), and nitrogen compound catabolic process (Fig. 1C, Additional file 3: Tab 4). In addition to these common terms, the clusters had distinctive functional enrichments, including ribosomal biogenesis in cluster 1, RNA splicing and processing in cluster 2, and chromatin modification and epigenetic silencing in cluster 3 (Fig. 1C, Additional file 3: Tabs 1–3, with Column E in each case listing the interactome proteins underlying each enriched ontology term, Additional file 5: Fig. S1).

Clustering and enrichment analyses were also conducted on the *RSX* and *Xist* interactomes separately using the same approach. Each interactome had four major clusters, with GSEA enrichments reflecting those of the combined interactome analysis, subject to division of cluster 2 in the *RSX* interactome, and division of cluster 1 in the *Xist* interactome (Additional file 5: Fig. S2).

*RSX*-specific interactome proteins were of interest in unravelling the differences between eutherian and marsupial XCI. GSEA of the 81 *RSX*-specific proteins identified enrichments for spliceosomal complexes, ribosomal subunits, cytosolic translation, nucleosome binding and chromatin organisation (Additional file 3: Tab 6) in proportions similar to those of the overall *RSX* interactome, other than perhaps for nucleosome binding which predominantly involves *RSX*-specific proteins. Apart from this, *RSX*-specific proteins did not appear to have gross unique function compared to the full *RSX* interactome.

Collectively, the clustering and GSEA enrichment analyses revealed an overlap between the *RSX* and *Xist* interactomes. This encompassed common proteins and also interactions with different proteins in common molecular pathways, providing insights into the functions modulated by *RSX* and *Xist.*

### Functional analysis of HNRNPK in *Monodelphis* XCI

We focused on the functional role of HNRNPK, which was identified in our *RSX* interactome and is also an *Xist*-interacting protein. HNRNPK is important in recruiting polycomb repressive complex 1 (PRC1), a significant part of the epigenetic silencing machinery, during eutherian XCI [11, 35]. In the combined *RSX/Xist* interactome network it was in cluster 2, which was enriched for functions in RNA splicing and processing (Fig. 1B). We depleted HNRNPK expression in a female *Monodelphis* fibroblast cell line using RNA interference (RNAi), adapting eutherian-based construct design and delivery for our non-traditional model organism. We assessed the effect on XCI using RNA FISH, which allowed us to determine the transcriptional status of *MSN,* an X-borne gene that is usually silenced on the inactive X chromosome, which should have monoallelic expression. In control nuclei (transfected with an empty RNAi vector) biallelic expression of *MSN* (indicating transcription from both X chromosomes) was detected in only 18% of cells (*n* = 286; Additional file 5: Figs. S3 and S4). Knockdown efficiency was assayed by measuring transcript abundance using RT-qPCR. The knockdown effect on protein abundance may differ due to variations in post-transcriptional processing.

Following HNRNPK knockdown (by ~ 24–35%) biallelic expression of *MSN* increased from 18% in control cells to 39% in cells with depleted HNRNPK expression (*n* = 159; *p* = 1.0 × 10^−11^ chi-squared test goodness of fit test) (Additional file 5: Figs. S3 and S4). Increased biallelic expression of *MSN* signified reactivation of transcription from the silenced allele on the inactive X chromosome. This outcome was observed across two independent experiments, and provides evidence that HNRNPK plays a role in maintenance of transcriptional silencing on the inactive X chromosome in *Monodelphis*.

### Functional analysis of CKAP4 in *Monodelphis* XCI

CKAP4 has not been identified as an *Xist* interactor, and has no predicted interactions with any protein in either the *Xist* or *RSX* interactomes (Additional file 4: Tab 3). In the *RSX* interactome, CKAP4 was unexpectedly the protein with the highest fold-change (20 ×) enrichment relative to controls in the native (uncrosslinked) ChIRP-MS (Additional file 2: Tab 2). Therefore, we used RNAi to suppress CKAP4 in female *Monodelphis* fibroblasts by ~ 53–55%. We observed an increase in biallelic expression of *MSN* from 18 to 26% (*n* = 165; *p* = 8.7 × 10^−3^ Chi Squared Test Goodness of Fit Test). This suggests that CKAP4 plays a role in maintenance of *Monodelphis* XCI.

Interestingly, despite the absence of CKAP4 from the GSEA analysis of the combined interactome network, the rough endoplasmic reticulum (where CKAP4 is usually localised) was significantly enriched in cluster 1 (*p* = 1.1 × 10^−6^), along with three other associated terms (*p* < 9.8 × 10^−4^) (Additional file 3: Tab 1). This finding aligns with the functional enrichment of ribosomal and translation-associated machinery observed in the same cluster.

**Fig. 2:**
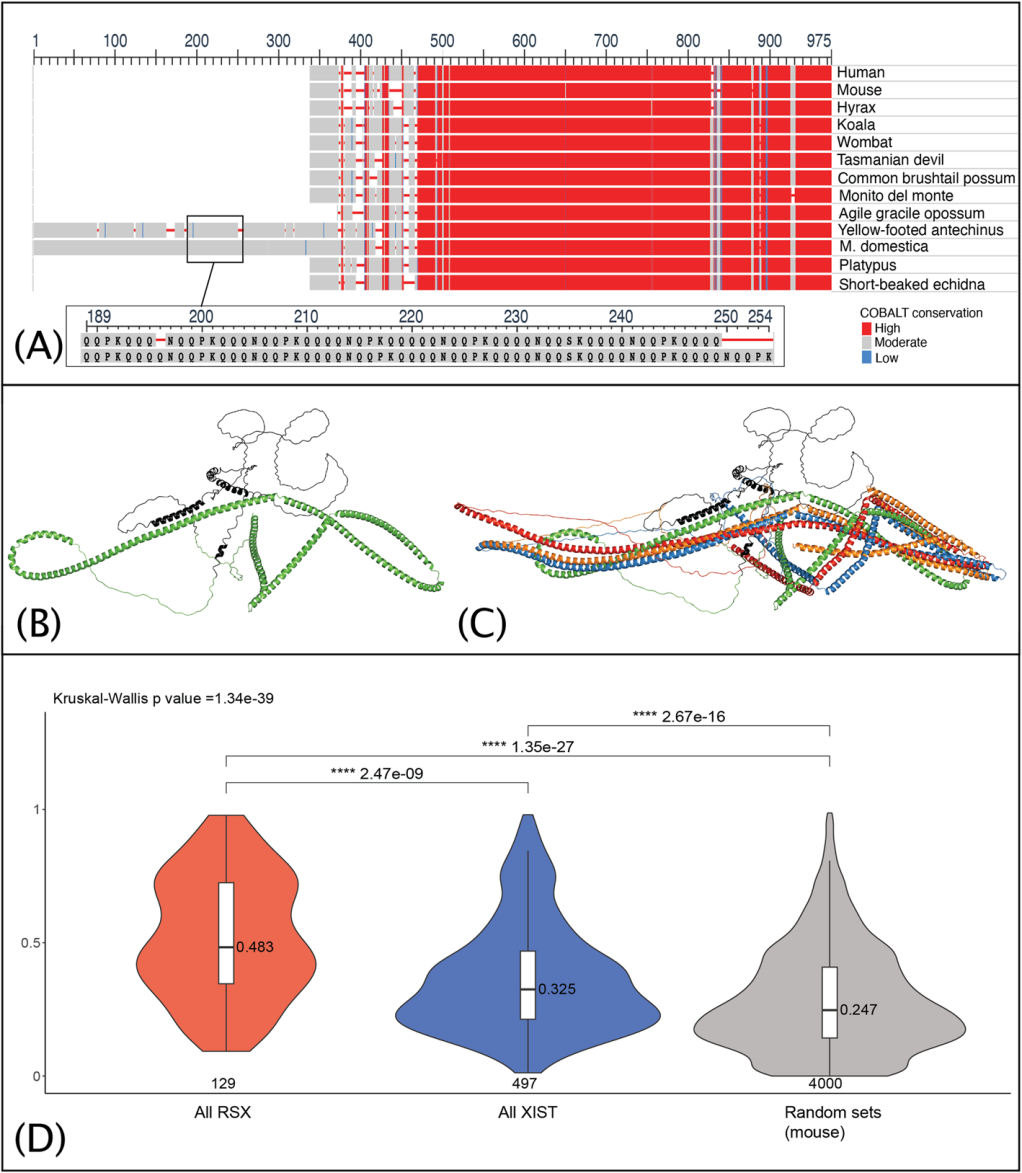
CKAP4 has a glutamine-rich repeat expansion in monodelphis. *RSX* and *Xist* interactomes are enriched for proteins with IDRs. **A** Protein sequence alignments of representative mammalian CKAP4. An expansion of a glutamine (Q) rich repeat was observed at the N-terminus in *Monodelphis* and yellow footed antechinus. Inset shows a subregion of the repeat expansion. **B** AlphaFold predicted structure of *Monodelphis* CKAP4, with the Q rich repeats highlighted in black. **C** Alignment of predicted CKAP4 structures for human (blue), mouse (red), hyrax (orange) and *Monodelphis* (green and black). Sequence independent RMSD values (for all atoms with outlier rejection) of *Monodelphis* CKAP4 to the eutherian orthologues were 24.5 Å (human), 24.5 Å (mouse), and 13.7 Å (hyrax). **D** Median protein IDR scores for the *RSX* and *Xist* interactomes represented as violin plots (depicting density distribution) overlayed with boxplots depicting the median for all proteins of the *RSX* and *Xist* interactomes (mouse orthologs), and randomly sampled proteins of a subset of the mouse proteome comprising only proteins within the gene ontology terms enriched in clusters 1, 2 and 3 (20 × sets of 200 proteins). Statistical significance assessed using Dunn’s test (with Holm adjustment) for pairwise comparisons, following Kruskal–Wallis test

The role of CKAP4 in marsupial XCI prompted a comparative analysis of its protein sequences across a broad phylogenetic spectrum, including eutherians (mouse, human, and hyrax — an afrotherian), monotremes (platypus and echidna), and eight marsupial species. This comparative sequence analysis (Fig. 2A), unveiled a large expansion of a glutamine (Q)-rich repeat at the N-terminus in the *Monodelphis* CKAP4, which contrasted eutherians, monotremes and most other marsupials.

We employed AlphaFold to predict the tertiary structures of CKAP4 in *Monodelphis* alongside three representative eutherian species: human, mouse, and hyrax (Fig. 2B and C). The structural predictions highlight a distinctive helical conformation within the Poly Q-rich N-terminus of *Monodelphis* CKAP4 (2B). Such structural motifs are known for their stability and propensity to engage in functional interactions with RNA and proteins [36], and provides a molecular mechanism by which *Monodelphis* CKAP4 could interact with *RSX*.

### Enrichment of proteins with intrinsically disordered regions (IDRs) in the *RSX* interactome

Recent studies have revealed that the *Xist* compartment is founded on an assembly of dynamic RNP complexes comprising *Xist* RNA in association with the intrinsically disordered regions (IDRs) of *Xist*-interacting proteins, such as SPEN, PTBP1, MATR3, CELF1, and CIZ1 [37–40]. Of these, only PTBP1 was identified in the *RSX* interactome, so we considered whether other proteins with IDRs might also be present in the *RSX* interactome.

We assessed the proportion of proteins enriched for IDRs in each interactome with IUPred2A [41], which calculates a disorder score for each residue using amino acid composition and energy estimation. Disorder scores above 0.5 (range 0 to 1) correspond to disordered residues. We calculated the IDR score for each protein as the median disorder score of its residues. We found that *RSX* interactome proteins had higher median IDR scores than the *Xist* interactome proteins (*p* = 2.5 × 10^−9^). Both interactomes had higher median IDR scores (*p* = 1.4 × 10^−27^ for *RSX* and *p* = 2.7 × 10^−16^ for *Xist*) than a randomly sampled group of 200 proteins from a subset of the reference proteome (Fig. 2D). The background proteome subset comprised all proteins within the ontology terms (Additional file 3, Tabs 1–3, column A) enriched (*p* < 1 × 10^−3^) in one or more of the combined interactome clusters 1, 2 and 3. Proteins common to both the *RSX* and *Xist* interactomes had higher median IDR scores than proteins that interact exclusively with either *RSX* or *Xist* (*p* = 2.5 × 10^−2^ *cf RSX*, *p* = 1.4 × 10^−10^ *cf Xist*) (Additional file 5: Fig. S5A).

IDRs have important roles in supporting protein–protein interactions, protein-RNA interactions, and the formation of phase-separated condensates that form nuclear subcompartments [42–44]. Our finding suggests that an enrichment for proteins with IDRs may play a role in the formation of *RSX*-associated RNP complexes, aiding subcellular organisation, as has been identified for *Xist*-associated RNPs.

## Discussion

This research provides a novel insight into the complex protein interactions of *RSX*, a lncRNA in marsupials with a role similar to the eutherian *Xist* in epigenetically silencing the inactive X chromosome. We found that that the *RSX* interactome has functional enrichments analogous to *Xist* that underscore their functional similarities. We also showed that alleles on the inactive X chromosome were partially reactivated following the partial depletion of HNRNPK and CKAP4, two proteins in the *RSX* interactome, indicating a role for each in marsupial XCI maintenance.

Of note was the glutamine (Q)-rich repeat at the N-terminus of CKAP4. Poly-Q motifs play a pivotal role in modulating protein–protein interactions, often leading to the formation of aggregates with distinct biological consequences [36]. The structure of the Poly-Q rich repeat in *Monodelphis* CKAP4 suggest a novel change that might underpin its different function when compared to the eutherian counterpart. The helical conformation of *Monodelphis* CKAP4 N-terminus could enhance affinity for RNA and/or proteins, which could enable its binding with *RSX*. Interestingly, the Poly-Q motif expansion is not common to all marsupials, appearing to be specific to *Monodelphis* and a species of antechinus, suggesting linage specific adaptation.

LncRNAs provide an important organising mechanism in epigenetic regulation, including in the recruitment and sequestration of RNA splicing and processing factors. Many of these proteins are multifunctional, often with distinct nuclear and cytoplasmic functions. This interaction of lncRNAs with multifunctional proteins can provide an efficient mechanism by which lncRNAs can impact diverse molecular networks. Of the 54 proteins identified in common in the *RSX* and *Xist* interactomes, 43 form part of interactome network cluster 2, which features proteins involved in RNA splicing and processing.

The 54 proteins common to both interactomes were enriched for intrinsically disordered regions (IDRs), a characteristic identified in each interactome individually. These IDRs could potentially contribute to epigenetic silencing by facilitating protein–protein interactions, as proteins enriched in IDRs are characterised by their flexible and adaptable binding with multiple partners. This binding plasticity may contribute to the dynamic regulation of gene expression on the inactive X chromosome, potentially including alternate silencing and escape from silencing, depending on specific cellular contexts. Further, this plasticity might be important for the observed ‘leakiness’ of XCI observed in marsupials as partial or full expression from the inactive X chromosome [45, 46].

Analysis of interactome network clusters 1 and 3 provided further insight into the mechanisms by which *RSX* and *Xist* might regulate gene expression. GSEA of each of these clusters indicated a functional coherence: despite a relatively small overlap in interactomes, they had different protein interactors involved in shared pathways. Cluster 1 was functionally enriched for ribosomal biogenesis, rRNA processing and regulation of translation. For *RSX*, this is consistent with the nucleolar association of the inactive X in marsupials. Cluster 3 contained proteins typically associated with XCI, including those involved in epigenetic regulation of transcriptional silencing, histone modifications and heterochromatin. These proteins include SPEN, which was identified in all of the *Xist* ChiRP-MS studies but was absent from the *RSX* interactome. SPEN is required for upregulation of *Xist* during XCI initiation [47], but becomes less important during the maintenance stage [48]. Therefore, it was not unexpected that SPEN was absent from the RSX interactome in our fibroblast model, which represents XCI maintenance.

Enrichment of functions associated with post-transcriptional regulation is a fascinating aspect of the *Xist* and *RSX* interactomes. Post-transcriptional regulation of X-borne gene expression has been identified in eutherians and *Monodelphis* by comparing gene expression in the transcriptome and translatome (based on ribosomal occupancy) [24]. Balancing of sex chromosome-borne gene expression between sexes in the proteome has also been identified in the more distantly related platypus (*Ornithorhynchus anatinus*) and chicken (*Gallus gallus*). This highlights the possibility of post-transcriptional regulation being an ancestral strategy for fine tuning the expression of sex chromosome genes in both sexes [49]. Our understanding of the evolution of the therian sex chromosomes suggests that silencing of X-borne genes would have evolved as the X and Y chromosomes diverged, perhaps initially involving other noncoding RNAs and only localised silencing before the Y chromosome was as degraded as it currently is. The emergence of independent chromosome wide regulation of XCI by *XIST* and *RSX* would then have coordinated presumably more efficient silencing, balancing the expression of X-borne genes between the sexes as degeneration of the Y chromosome progressed.

## Conclusions

This work highlights a striking example of convergent evolution of lncRNA protein interactome evolution that achieves XCI in diverse mammalian clades. The independently evolved *XIST* and *RSX* recruit similar molecular pathways to repress the activity of almost an entire chromosome. These molecular pathways are associated with epigenetic transcriptional silencing, which typifies XCI, in addition to post-transcriptional regulation of gene expression, notably RNA splicing and processing, translation regulation and ribosome biogenesis. The functional coherence between the *RSX* and *Xist* interactomes, and the prevalence in both interactomes of proteins enriched for IDRs, adds a novel and critical dimension to our understanding of lncRNA mediated epigenetic regulation.

## Methods

### Cell culture

Female *Monodelphis* fibroblasts were cultured at 35 °C with 5% CO_2_ in Dulbecco’s modified Eagle’s medium (DMEM), 10% v/v Newborn Calf Serum, 0–10% v/v AmnioMAX™-C100. Cells were passaged at 70–100% confluency using Trypsin–EDTA (0.25% w/v) (Thermo Fisher Scientific).

### ChIRP-MS

Cells were cultures on 15-cm plates to 70–80% confluence. Three plates (~ 6 µg total protein) were used for each pulldown. Cross-linking of samples occurred prior to cell harvest using either: (1) UV using Stratalinker UVP Crosslinker CL-1000 (200 mJ/cm^2^ at 200 nm) on ice in 10 ml of phosphate buffered saline (PBS); or (2) 3% formaldehyde solution in PBS (30 min, RT) followed by quench in 0.125 M glycine for 5 min. ‘Native’ samples were not cross-linked. Cells were scraped from the plate into Eppendorf tubes and pelleted at 500 rcf for 5 min at 4 °C. Cell pellets were alternately flash-frozen and stored at – 80 °C, or proceeded directly. The cell pellet was resuspended (1 ml per plate of cells) in NP-40 buffer with Roche cOmplete™, EDTA-free Protease Inhibitor Cocktail (1 tablet per 7 ml of NP-40 buffer) and incubated for 15 min either: 1) at 4 °C on rotating platform; or) on ice, with vortexing for 5 s every 5 min. Cell lysates were sonicated using a Q700 sonicator (Qsonica) in a 4 °C water bath at amplitude 16 for 12 min pulsing 30 s on/off. Sonicated cell lysates were pelleted at 20,000 rcf for 5 min at 4 °C. Supernatant lysate was removed and assayed for total protein concentration using a Qubit™ fluorometer. Aliquots of 1.5 ml of supernatant (~ 3 µg total protein) were combined with 50 µl of prepared Dynabeads™ M-280 Streptavadin (beads). Beads were prepared in accordance with manufacturer’s guidelines with the following modifications: 100 µl of resuspended beads were used for each sample. After washing in 1 × Binding + Wash buffer (10 mM Tris–HCl (pH 7.5) 1 mM EDTA 2 M NaCl), beads were washed twice in Solution A (DEPC-treated 0.1 M NaOH DEPC-treated 0.05 M NaCl) followed by twice in Solution B (DEPC-treated 0.1 M NaCl), in each case vortexing for 5 s before magnetic capture for one min. Suspended beads were divided into 100 µl aliquots before final magnetic capture, followed by addition to each aliquot 200 µl of 2 × B + W buffer, 5 µl of 100 mM biotin-labelled oligonucleotide (omitted for control) and 195 µl of DNase-free H_2_O. Samples were incubated for 15 min at room temperature on slow rotating platform. Beads were magnetically captured for 3–5 min, before removal of clear supernatant. Beads were washed 3 times with a 1 × B + W buffer (200 µl) on rotation for 2 min with a one min magnetic capture. Beads were re-suspended in 100 µl of NP40 Buffer, before dividing between the two 1.5-ml aliquots of supernatant for each sample. Lysates were incubated with the pre-cleared beads on a rotating platform overnight at 4 °C. Beads were magnetically captured for one min at 4 °C, and supernatant removed. The beads for each sample were then washed with twice with 2 ml of NP40 Buffer (divided equally between the bead aliquots for the first wash before combining for the second wash), followed by twice for 15 min with 1 ml RIPA Buffer on rotating platform at 4 °C. After the final magnetic capture, the supernatant was removed. One hundred microliters of pre-warmed (65 °C) Elution Buffer (10 mM Tris–HCl, pH 6.0, 1 mM EDTA, 2.0M NaCl) was added to the supernatant before incubating for 20 at 65 °C with shaking. The supernatant was magnetically cleared of beads twice before assaying the protein concentration of the supernatant using a Qubit™ fluorometer, and submitting for LC/MS–MS. For validation, supernatant containing 20–60 μg of protein was dissolved in 1 × Laemmli Buffer (Bio-Rad), heated to 95 °C for 10 min to denature, and then size-separated on a 7.5% SDS-PAGE gel (Bio-Rad TGX) in 1 × Tris/Glycine/SDS (TGS) Buffer (Bio-Rad) at 160 V. Protein gels were washed in Milli-Q water three times for 5–10 min each before staining overnight in Commassie blue, washing in RO water three times and then excising protein bands.

### Probe design

ChIRP-MS probes (Additional file 1) were designed using online tools [50]. Oligonucleotide probes were synthesised with 3′ Biotin-TEG, obtained from Integrated Data Technologies, Inc.

For each pull-down oligonucleotide probes were either pooled, or used individually. Probe 3 targeted the *RSX* Repeat 1. A probe with no homology to any sequence in the *Monodelphis* genome was used as an additional control to filter proteins identified in native (uncrosslinked) pull-downs.

### Mass spectrometry

Samples were analysed at the Bioanalytical Mass Spectrometry Facility at the Mark Wainwright Analytical Centre (UNSW, Australia). Briefly, samples were firstly buffer exchanged to ammonium bicarbonate via 3 kDa spin cartridge. Samples were reduced (5 mM DTT, 37 °C, 30 min), alkylated (10 mM iodoacetamide, RT, 30 min), and incubated with trypsin at 37 °C for 18 h, at a 1:20 ratio (w/w). Samples were desalted with 200 µl C18 stage tip tips (Thermo Fisher Scientific). Eluted peptides from each clean-up were reconstituted in 10 µL 0.1% (v/v) formic acid and 0.05% (v/v) heptafluorobutyric acid in water. Digest peptides were separated by nano-LC using an Ultimate 3000 HPLC and autosampler system (Dionex, Amsterdam, Netherlands). Samples (2.5 µl) were concentrated and desalted onto a micro C18 precolumn (300 µm × 5 mm, Dionex) with H2O:CH3CN (98:2, 0.05% TFA) at 15 µl/min. After a 4 min wash the pre-column was switched (Valco 10 port valve, Dionex) into line with a fritless nano column (75µ ×  ~ 10 cm) containing C18 media (1.9 µ, 120 Å, Dr Maisch, Ammerbuch-Entringen Germany) manufactured according to Gatlin [51]. Peptides were eluted using a linear gradient of H2O:CH3CN (98:2, 0.1% formic acid) to H2O:CH3CN (64:36, 0.1% formic acid) at 200 nl/min over 30 min. High voltage 2000 V) was applied to low volume tee (Upchurch Scientific) and the column tip positioned ~ 0.5 cm from the heated capillary (*T* = 275 °C) of an Orbitrap Velos ETD (Thermo Electron, Bremen, Germany) mass spectrometer. Positive ions were generated by electrospray and the Orbitrap operated in data dependent acquisition mode (DDA).

A survey scan m/z 350–1750 was acquired in the Orbitrap (resolution = 30,000 at m/z 400, with an accumulation target value of 1,000,000 ions) with lockmass enabled. Up to the 10 most abundant ions (> 4000 counts) with charge states >  + 2 were sequentially isolated and fragmented within the linear ion trap using collisionally induced dissociation with an activation *q* = 0.25 and activation time of 10 ms at a target value of 30,000 ions. M/z ratios selected for MS/ MS were dynamically excluded for 30 s.

LC–MS/MS spectra were analysed using the MaxQuant software suite (version 1.6.2.10.43) [52]. Sequence database searches were performed using Andromeda [53]. Label-free protein quantification was performed using the MaxLFQ algorithm [54]. Delayed normalizations were performed following sequence database searching of all samples with tolerances set to ± 4.5 ppm for precursor ions and ± 0.5 Da for peptide fragments. Additional search parameters were: carbamidomethyl (C) as a fixed modification; oxidation (M) and N-terminal protein acetylation as variable modifications; and enzyme specificity was trypsin with up to two missed cleavages. Peaks were searched against the reference genome for *Monodelphis* (Ensembl release 97). MaxLFQ analyses were performed using default parameters with “fast LFQ” enabled. Protein and peptide false discovery rate (FDR) thresholds were set at 1% and only non-contaminant proteins identified from ≥ 2 unique peptides were subjected to downstream analysis.

Protein groups files were imported into R Studio for analysis. Proteins were identified using a combination of (i) presence/absence analysis, to identify proteins detected in two or more pulldowns for native samples and a single pulldown for UV crosslinked samples and formaldehyde crosslinked; and (ii) intensity-based analysis, to identify proteins enriched more than threefold (log_2_ ratio > 1.584963) relative to a control. For the fold-change analysis for formaldehyde crosslinking and UV crosslinking, proteins were selected on this basis alone. For the fold-change analysis for native (no crosslinking), proteins were subject to additional filtering: (i) proteins detected by more than 4 of 6 different ChIRP probe combinations (comprising 5 *RSX* probes individually + all *RSX* probes together); and (ii) *t*-test (with Benjamini–Hochberg correction for multiple testing), *p* value < 0.05.

### RNA immunoprecipitation (RIP)

The RIP was performed on female *M.domestica* cells as described in [21] using the antibodies set out in Additional file 1. Quantitative RT-qPCR was performed using *RSX*-specific primers as described in [21].

### RNAi knockdown

The shRNA-expressing constructs were designed using a combination of online tools [55–57], and nucleotide BLAST of candidate sequences against the MonDom5 genome assembly (Ensembl release 84). For each target mRNA, candidate constructs were trialled for different regions of the mRNA to accommodate the possibility that binding may be impeded at certain sites by secondary structure or sequence variants. Constructs were cloned into a pCDNA3-U6M2 plasmid vector using BglII and KnpI restriction sites as described previously [58]. In summary, 2 ug of vector DNA was digested with KpnI-HF in 1 × CutSmart® buffer, then with BglII in 1 × NEB buffer 3.1. Each digest was incubated in total volume of 50 μl (including BSA 5 μl, 1 mg/ml) at 37 °C for one hour. The vector DNA was purified after each digest using the QIAquick® PCR Purification Kit. The vector was then 5′ dephosphorylated using Antarctic Phosphatase. The shRNA construct was prepared by 5′ phosphorylation of the oligonucleotides with T4 Polynucleotide Kinase, followed by annealing of the complementary oligonucleotides at 95 °C for 5 min, cooled to 25 °C over 1 h. The cut vector and shRNA construct were ligated with T4 DNA Ligase and transformed into competent *Escherichia coli* DH5α cells. Five microliters of vector (10–15 ng) was added to 50 μl cells, incubated on ice for one hour, heat-shocked at 42 °C, for 45 s, then incubated in 350 μl SOC medium at 37 °C for one hour. Competent DH5α cells were prepared by culturing in Luria Broth at 37 °C to optical density A_600_, then incubating on ice for 10 min, pelleting by centrifugation at 1520 rcf for 10 min at 4 °C, resuspending in Transformation buffer (6 mL), before storing at − 80 °C. Transformed DH5α cells were plated on ampicillin selective Luria Both agar. Gel electrophoresis of colony PCR product was used to screen for colonies cloned with the shRNA template. PCR reactions were performed with primers p008 and p080 using Taq 2 × Mastermix according to manufacturer’s instructions. PCR products of candidate clones were then sequenced using BigDye v.3.1 by the Ramaciotti Centre for Genomics (UNSW Sydney, Australia) to confirm cloning accuracy. Successful clones were cultured overnight at 37 °C in selective Luria Broth (ampicillin 100 μg/ml), and then extracted using the QIAGEN® Plasmid Midi Kit according to manufacturer’s instructions.

### Transfection

The shRNA vector plasmids were introduced into the *Monodelphis* fibroblasts by transfection with Lipofectamine 3000. First transfection was carried out when cells were at 70–80% confluency. For transfection of cells on coverslips in 6-well plate, vector DNA (2.5 μg), Lipofectamine® 3000 reagent (6.5 μl) and P3000® reagent (5 μl) diluted in 250 μl Opti-MEM medium, added to 1.5 ml of cell culture medium. For transfection of cells in T25 flask, vector DNA (7.5 μg), Lipofectamine® 3000 reagent (18 μl) and P3000® reagent (15 μl) diluted in 375 μl Opti-MEM medium, added to 4 ml of cell culture medium. A second transfection was carried out ~ 24 h after initial transfection. For each transfection, incubations were conducted as per manufacturer’s instructions and cell culture media was replaced 6 h after transfection. Cells were harvested for RNA extraction, or progressed to RNA FISH, ~ 24 h after the second transfection.

### RNA extraction

RNA was extracted from transfected cells using TRIzol® reagent (Invitrogen), 1.5 ml for T25, 500 µl for per well of 6-well plate, with incubation at room temperature for 5 min with mild agitation. Chloroform was added (0.2 ml chloroform per 1 ml of TRIzol® reagent), vortexed and incubated for 10–15 min at room temperature, before centrifuging at 10,000 rcf for 15 min at 4°. The aqueous (upper) phase was aspirated, and 1.5 × volume of 100% ethanol was slowly added and mixed. RNA was purified from the sample using RNeasy spin column kit (according to manufacturer’s instructions and on-column DNAse digestion using the RNase-free DNase set according to the manufacturer’s instructions. Final elution of RNA was in Ultra-Pure™ DEPC-treated water. RNA concentration was assayed using Qubit™ RNA Assay.

### shRNA knockdown RT-qPCR

cDNA was prepared using Superscript™ IV First-Strand Synthesis System according to the manufacturer’s instructions, with 70–300 ng of total RNA as template and using oligo dT primers. RT-qPCR was conducted using the Viia7 Real-Time PCR System (Applied Biosystems) in technical triplicate using the KAPA SYBR® FAST qPCR Kit Mastermix (2 ×) Universal, using 0.5 μl of cDNA template and gene-specific primers (10 μM) (Additional file 1) in a 10-μl reaction. PCR was conducted at 95 °C for 20 s for enzyme activation, followed by 40 cycles of: 95 °C (1 s), 60 °C (20 s), then 60 °C to 99 °C melt curve analysis. Target expression level was calculated using the ΔΔCt method relative to the control cells transfected with an empty pCDNA3-U6M2 plasmid vector, and with normalisation with reference to GAPDH.

### RNA FISH probe preparation

RNA FISH probes were derived from BAC clones from the VMRC-18 BAC library (CHORI BACPAC Resources Centre (Oakland, CA)). Msn (BAC clone VM18-777F) was identified as a highly expressed X-linked gene based on RNA-seq transcriptome data (unpublished) from the same female *Monodelphis* fibroblasts, and mapping using Ensembl monDom5 assembly (release 84). The BAC clone containing *RSX* (VM18-839J22) was previously identified [18]. The BAC clones were acquired in *E. coli* DH10B, cultured in selective Luria Broth (chloramphenicol, 34 mg/ml) and extracted with the QIAGEN® Large Construct Kit. The BAC DNA was labelled by nick translation using DNase1, DNA Polymerase 1, fluorescent dUTP (0.03 mM Green 496 dUTP or Orange 552 dUTP) and nick translation buffer, with incubation at 15 °C for 1.5 h. Labelled probes were filtered through a sephadex column to remove unincorporated nucleotides. Probe size (~ 200–700 bp) was verified using gel electrophoresis (1% agarose). The labelled probes (100–200 ng per coverslip) were co-precipitated overnight at − 80 °C with *Monodelphis* C_0_t-1 DNA, and 100% ethanol v/v (3 × volume). After centrifugation (18,000 rcf, 4 °C, 30 min), the probe was washed twice in 70% ethanol, then air dried, dissolved in formamide (5 μl per coverslip, UNILAB), then denatured (75 °C, 7 min). The probe was then combined with hybridization buffer (5 μl per coverslip) and incubated on ice for 5 min, then at 37 °C for 20 min.

### RNA FISH

Sterilised coverslips were coated with gelatin before seeding in a 6-well plate with cells to density of ~ 70% confluence in overnight culture. Cells were washed with 1 × PBS before being permeabilized with Cytoskeletal buffer on ice for 5–7 min, then fixed in freshly made paraformaldehyde (3% w/v in 1 × PBS) for 10 min at room temperature, washed twice in 70% ethanol for 5 min, then dehydrated in an ethanol series (80%, 95%, 100% each for 3 min) before air drying. The prepared coverslip and probe (10 μl per coverslip) were hybridised on an RNase-free glass slide, sealed with vulcanised rubber, incubated overnight at 37 °C in a chamber, humidified with tissues soaked in 5 ml of 50% formamide/2 × SSC. Coverslips were then washed in a solution of formamide 50% v/v/2 × SSC (3 washes, each at 42 °C, 5 min), and then in 2 × SSC (3 washes, each at 42 °C, 5 min), air-dried and mounted. Coverslips were mounted with Prolong™ Diamond AntiFade Mountant with DAPI and sealed with clear nailpolish. Prepared slides were analysed using an Olympus BX53 microscope with proprietary cellSens software. Images were processed and compiled using Fiji (ImageJ) [59].

### Interactome analysis

The *Xist* and *RSX* interactome analysis was conducted using the STRING database (v 11.5) [28] using human as the reference species. Human orthologues of *Monodelphis* genes were identified using Ensembl 97 BioMart [60]. For genes without a one-to-one orthologue, a human orthologue or equivalent was identified using reciprocal protein BLASTs [61]. There were two exceptions to this, where the MonDom5 gene did not have a human 1 to 1 orthologue (ENSMODG00000025105), or where the best reciprocal blast hit was already represented in the *RSX* interactome (ENSMODG00000013903). In these cases, the gene was excluded from downstream analysis. Evidence of interaction was based on experimentally determined interactions, curated database annotations, and experimentally determined co-expression. Minimum interaction confidence was set at 0.400 (calculated on a scale of 0 to 1). Interaction networks were visualised using Cytoscape (v 3.8.2) [29], omitting proteins with no interactions. Clusters were generated using the GLay Cytoscape plugin [30] with prefuse force directed layout. GSEA was conducted using gProfiler2 (v 0.2.2) [31] for annotations GO:MF, GO:CC, GO:BP (BioMart classes releases 2023–03-06) in R Studio with multiple testing correction based on false discovery rate and filtering for ontology term size < 1600.

### Intrinsically disordered region analysis

The IDR content in interactome proteins was assessed using IUPred2A [41] using the idpr package (v 1.12.0) in R Studio using UniProt Accession ids (release 2023_02). This calculated a disorder propensity score for each residue based on parameters designed to detect long IDRs (at least 30 consecutive residues), with scores ranging from 0 to 1 and a score over 0.5 indicating a disordered residue. The median IDR score for each protein was calculated as the median of the per residue disorder scores.

Random sets (mouse and *Monodelphis* orthologs) were generated by randomly sampling (20 sets of 200 proteins each) from subsets of the UniProt (release 2023_02) proteomes UP000000589 (Mus musculus, Organism ID 10090) and UP000002280 (Monodelphis, Organism ID 13616), respectively, using the set.seed() function in R (version 4.1.3). The background proteome subset comprised all proteins within the ontology terms (Additional file 3, Tabs 1–3, column A) enriched (*p* < 1 × 10^−3^) in one or more of the combined interactome clusters 1, 2 and 3. Gene sets were obtained for each species from the GMT files for each of GO:BP, GO:MF, GO:CC [62]. Mouse orthologues of proteins in the *RSX* interactome were identified using Ensembl 97 BioMart [60].

For genes without an identified one-to-one orthologue, a mouse orthologue was identified using reciprocal best hit protein BLAST [61]. Three *RSX* interactors were not represented in the mouse orthologue set, either because no 1 to 1 orthologue was identified (ENSMODG00000006291, ENSMODG00000024476), or where the best reciprocal BLAST hit was already in the *RSX* interactome (ENSMODG00000008362). The statistical difference between groups was determined using the Kruskal-Wallace test followed by Dunn’s test (with Holm adjustment) for pairwise comparisons.

### CKAP4 protein sequence alignment and structure prediction

We retrieved CKAP4 orthologues sequences from the NCBI Gene database [63] and Ensembl (v 111) [64]. The selected orthologues included representatives from marsupials, monotremes and eutherians (human, mouse) model organisms and an Afrotheria species (rock hyrax), aiming to encompass a broad phylogenetic spectrum. The specific protein accession numbers selected for this analysis were ENSP00000367265 (human); ENSMUSP00000050336 (mouse); ENSPCAP00000004540 (rock hydra); XP_020838076.1 (koala); XP_027728405.1 (wombat); XP_031793680.1 (Tasmanian devil); XP_036617112.1 (common brushtail possum); XP_043823845.1 (monito del monte); XP_044535072.1 (agile gracile opossum); XP_051817421.1 (yellow-footed antechinus); ENSMODP00000002342 (monodelphis); XP_028935129.1 (platypus); XP_038612756.1 (short-beaked echidna). Alignment was performed using the NCBI Multiple Sequence Alignment Viewer (v 1.25.0, COBALT) [65] with default settings.

For structural predictions, we used Colabfold v1.4.0 running on the Gadi supercomputer system at the National Computational Infrastructure (NCI), Canberra, Australia. This approach leverages the predictive power of AlphaFold2, incorporating both template-based and template-free modelling to predict protein structures with high accuracy. The FASTA sequence files for CKAP4 from human, mouse, hyrax, and *Monodelphis* were inputted for structural prediction. Default parameters were used for the database search. To ensure robustness of predictions, a recycle count of 3 was used, enhancing the iterative refinement of the predicted structures. Furthermore, we employed the –amber flag to incorporate molecular dynamics simulations for refining the predicted structures, and the –templates flag to utilise available structural templates that could guide the folding prediction.

### Supplementary Information

Additional file 1: Table of reagents, resources and oligonucleotides.

Additional file 2: Tab 1. RSX and Xist interactomes. Tab 2. ChIRP-MS results.

Additional file 3: Tabs 1 - 3. GSEA analysis of combined interactomes network clusters 1 – 3, respectively. Column E (‘Genes’) lists the proteins in each enriched gene ontology term for the interactomes. Tab 4. GSEA terms enriched in all of combined interactomes network clusters 1-3. Tab 5. GSEA analysis of all proteins in each of RSX and Xist interactomes analysed separately (i.e., not combined) and including proteins with no predicted protein-protein interactions). Columns G and J (‘Genes.Rsx’ and ‘Genes.Xist’) list the interactome proteins underlying each enriched gene ontology term in the RSX and Xist interactomes, respectively. Tab 6. GSEA analysis of all proteins present in RSX interactome and absent from Xist interactome. Column E (‘Genes’) lists the interactome proteins in each enriched gene ontology term.

Additional file 4: Tab 1. Numbers of proteins and edges in STRING networks and each cluster of combined interactomes. Tab 2. Proteins in each cluster of combined interactomes. Tab 3. Proteins of combined interactomes with no predicted protein-protein interactions.

Additional file 5: Five additional supplementary figures and legends: Figure S1. Graphical abstract. Figure S2. Protein-protein association networks of Xist and RSX interactomes. Figure S3. RNA FISH images using probes for RSX and X-borne gene, MSN, following RNAi knockdown of HNRNPK and CKAP4. Figure S4. Additional RNA FISH images (control and HNRNPK RNAi knockdown). Figure S5. Median protein IDR scores for RSX and Xist interactomes.

Additional file 6: Review history.

## Data Availability

Mass spectrometry protein files and custom R scripts used to filter them are available via github.com [66]. RNA FISH images additional to Figures S3 and S4 are available via Figshare [67]. All other data are available in the manuscript or the supplementary materials.
