# Supplementary material for: Identification of the RSX interactome in a marsupial shows functional coherence with the Xist interactome during X inactivation
Source: Genome Biol. 2024 May 23;25:134. doi: 10.1186/s13059-024-03280-0 (PMC11112854; doi:10.1186/s13059-024-03280-0)
Supplement: Supplementary file 5 — Additional file 5: Five additional supplementary figures and legends: Figure S1. Graphical abstract. Figure S2. Protein-protein association networks of Xist and RSX interactomes. Figure S3. RNA FISH images using probes for RSX and X-borne gene, MSN, following RNAi knockdown of HNRNPK and CKAP4. Figure S4. Additional RNA FISH images (control and HNRNPK RNAi knockdown). Figure S5. Median protein IDR scores for RSX and Xist interactomes. [file 13059_2024_3280_MOESM5_ESM.pdf]

Additional file 5

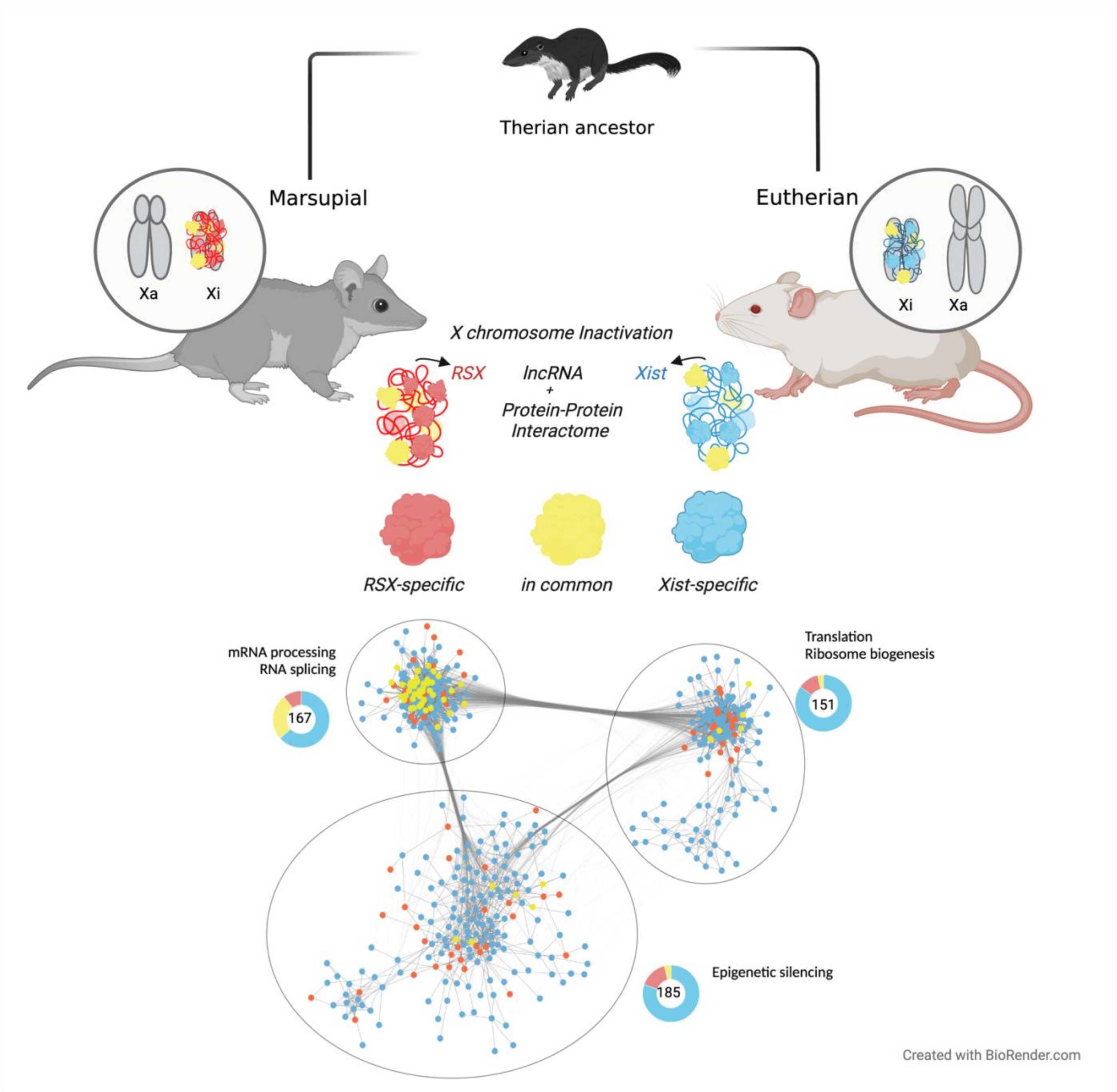

Figure S1. Graphical abstract.

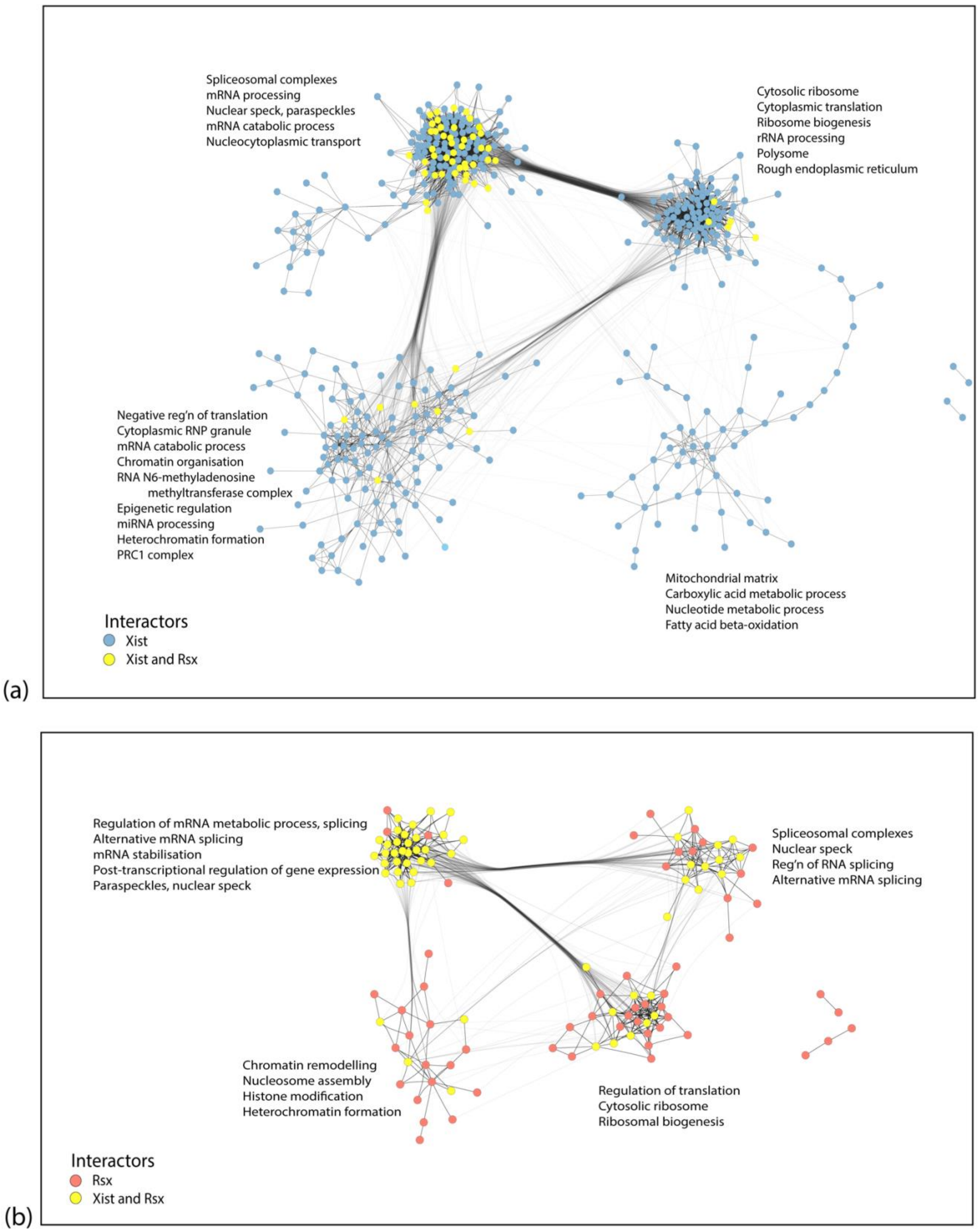

Figure S2. Protein-protein association networks of Xist and RSX interactomes.

Protein-protein interactions of the: **a)** *Xist*; and **b)** *RSX* interactomes, based on experimentally determined interactions, co-expression, and curated database annotations for human orthologs (STRING database v11.5) (29). Each node represents an interactome protein, each edge represents an annotated protein-protein interaction of minimum confidence 0.4. Interaction networks were visualised using Cytoscape (v 3.8.2), omitting proteins with no annotated interactions. Nodes were clustered based on connectivity (number and weight of edges) using the GLay Cytoscape plugin (47) with default settings (prefuse force directed layout).

Heavy edges represent intracluster protein-protein interactions, and light edges represent intercluster interactions. Selected functional and structural enrichments ($p < 1 \times 10^{-3}$) of each major protein interaction cluster were annotated, based on GSEA was conducted using gProfiler2 (48) in R Studio with multiple testing correction based on false discovery rate.

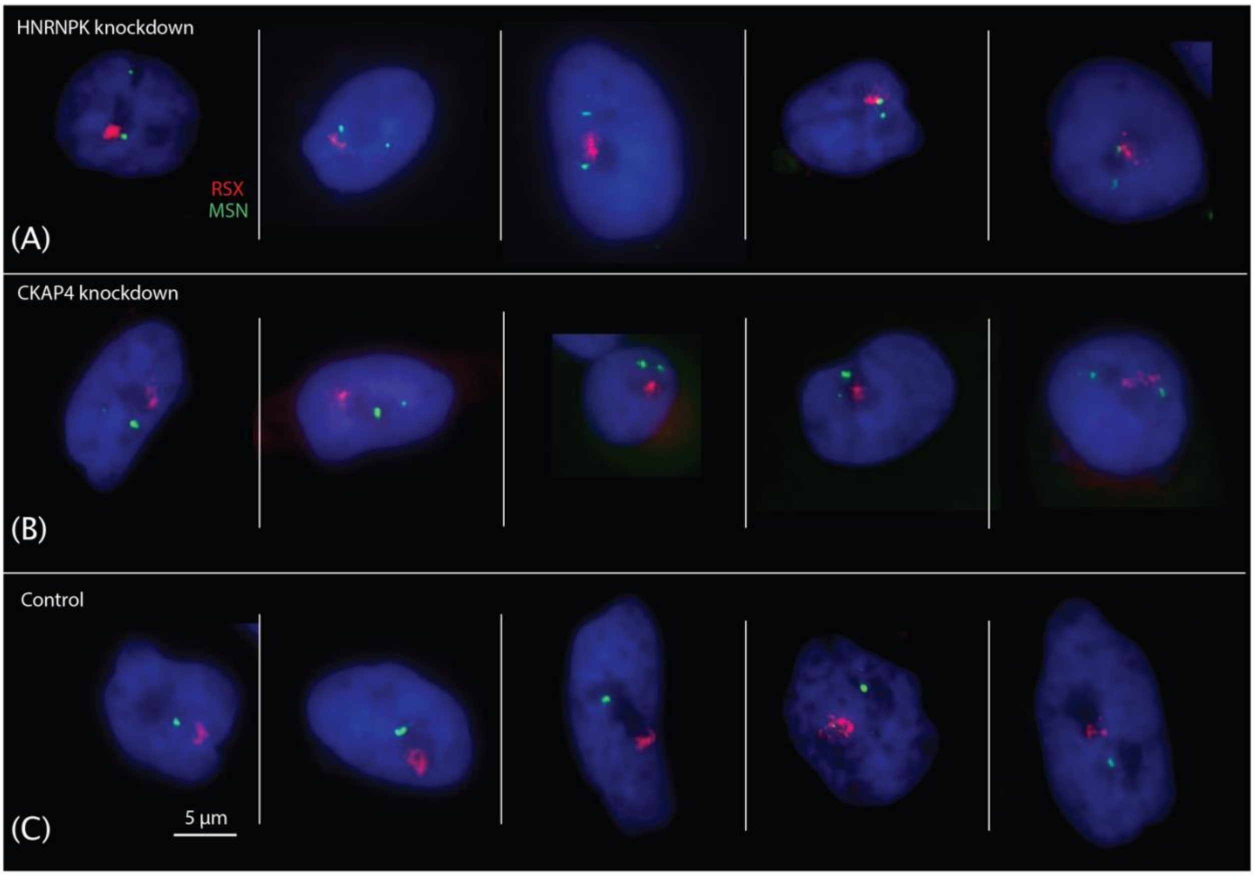

Figure S3. HNRNPK and CKAP4 have roles in maintaining XCI. Dual color RNA FISH using probes for *RSX* (red), and the X-borne gene, *MSN* (green), in female *Monodelphis* fibroblasts, representative images. Scale bar applies to all panels **(A)** RNAi knockdown of the RSX-interacting protein, HNRNPK (~24-35% knockdown efficiency) led to reactivation of transcription from the inactive X chromosome, evident as two *MSN* signals ($p = 1.0 \times 10^{-11}$ Chi Squared Test Goodness of Fit Test, n = 159). **(B)** RNAi knockdown of the RSX-interacting protein, CKAP4 (~53-57% knockdown efficiency) led to reactivation of transcription from the inactive X chromosome, evident as two *MSN* signals ($p = 8.7 \times 10^{-3}$ Chi Squared Test Goodness of Fit Test, n = 165). **(C)** Control nuclei transfected with empty plasmid vector typically have monoallelic expression of *MSN* that yields a single (green) signal per nucleus.

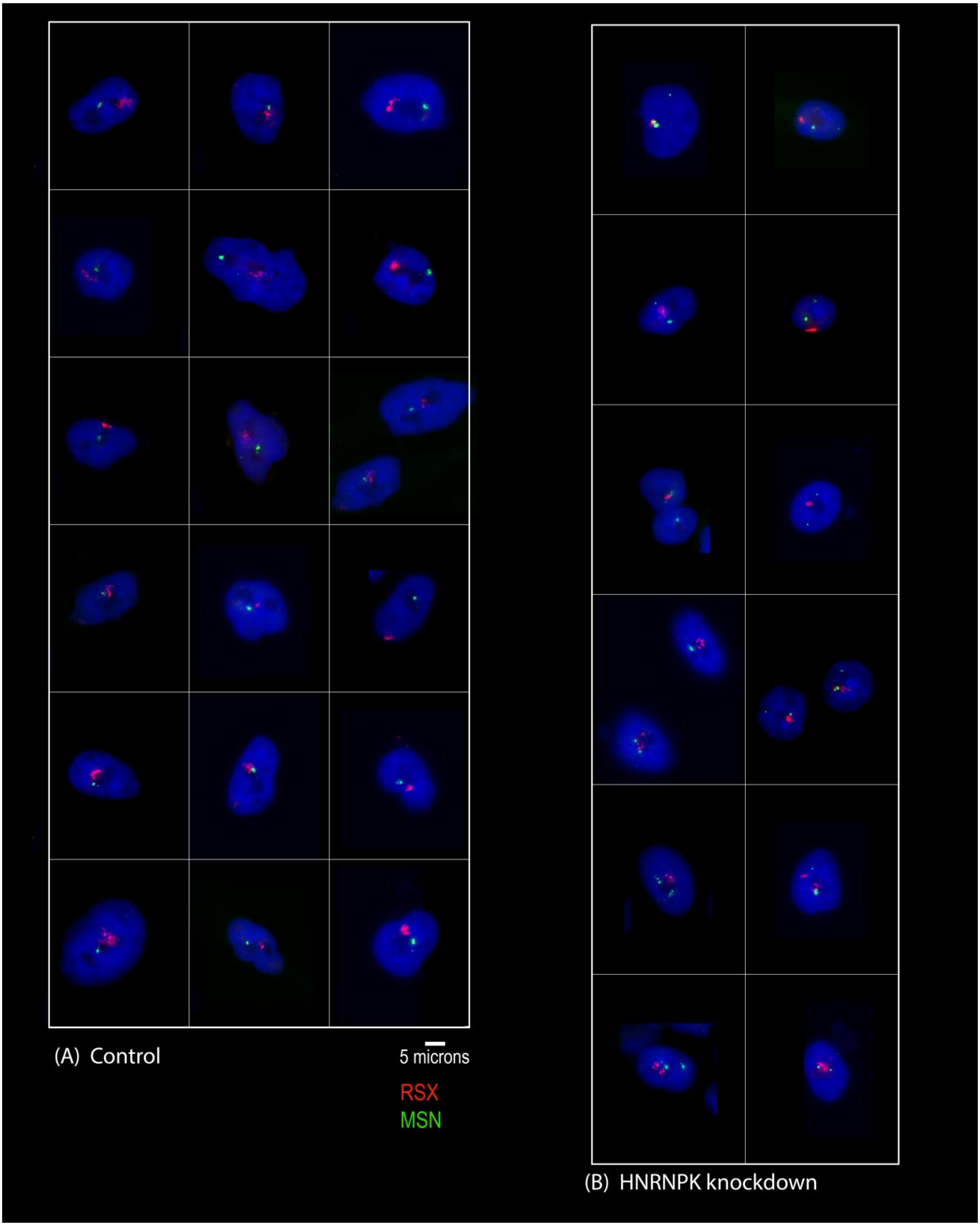

Figure S4. Additional RNA FISH images (control and HNRNPK knockdown).

Dual color RNA FISH using probes for *RSX* (red) and X-borne gene, *MSN* (green) in female *M. domestica* fibroblasts, selected images further to Figure 3A. **a)** Control nuclei. **b)** RNAi knockdown of HNRNPK (~24-35% knockdown efficiency).

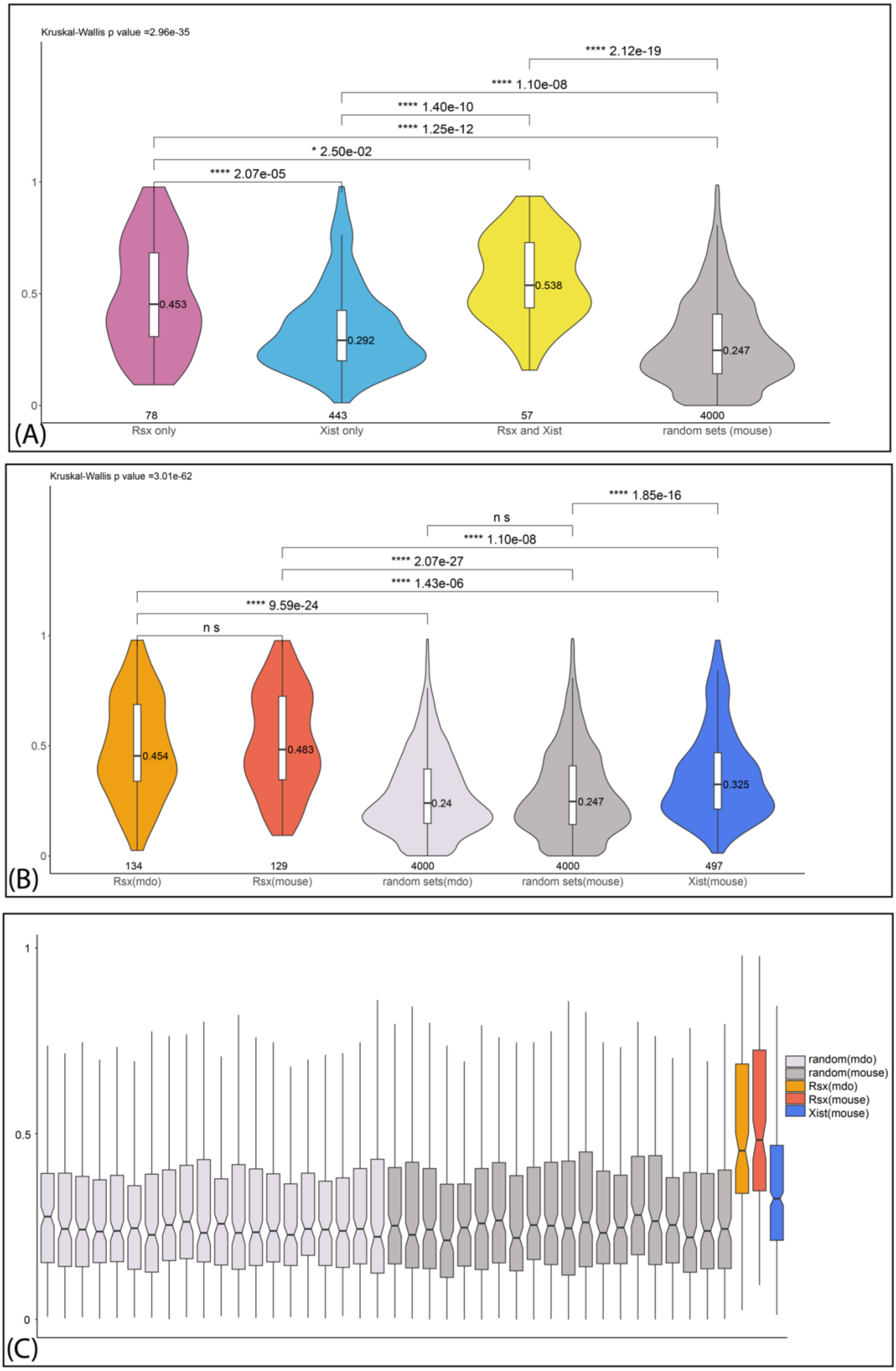

Figure S5. Median protein IDR scores for *RSX* and *Xist* interactomes

Median protein IDR scores represented as violin plots (depicting density distribution) overlayed with boxplots depicting the median for: **A)** mouse orthologs of proteins common to the *RSX* and *Xist*

interactomes, proteins present only in the *RSX* or the *Xist* interactome, randomly sampled proteins of a subset* of the mouse proteome (20x sets of 20 proteins combined); **B)** all proteins of the RSX interactome (mouse and *M. domestica* orthologs), all proteins of the *Xist* interactome (mouse orthologs), randomly sampled proteins of a subset* of the mouse and *M. domestica* proteomes (20x sets of 200 proteins combined); **C)** sets of randomly sampled 200 proteins from subsets* of each of *M. domestica* and mouse proteomes, all proteins of the RSX interactome (*M. domestica* and mouse orthologs), all proteins of the *Xist* interactome (mouse orthologs). Statistical significance assessed using Dunn's Test (with Holm adjustment) for pairwise comparisons, following Kruskal-Wallis Test. * proteome subsets used for random sampling comprised all proteins identified with the genes supporting the annotation of the gene ontology terms enriched in interactome clusters 1, 2 or 3 in each species, respectively.
