# Supplementary material for: Identification of the RSX interactome in a marsupial shows functional coherence with the Xist interactome during X inactivation
Source: Genome Biol. 2024 May 23;25:134. doi: 10.1186/s13059-024-03280-0 (PMC11112854; doi:10.1186/s13059-024-03280-0)
Supplement: Supplementary file 6 — Additional file 6: Review history. [file 13059_2024_3280_MOESM6_ESM.docx]

**1^st^ round**

**Reviewer 1**

Mcintyre et al. explores the functional homology between RSX and Xist by a comparative analysis of the protein networks that interact with the two lncRNAs in marsupials and eutherian mammals. The RSX lncRNA silences one of the two X chromosomes in female marsupials, similarly to the Xist lncRNA that controls X chromosome inactivation in female mice. There is, however, no sequence homology between the RSX and Xist genes, implying convergent evolution based on functional homology specifically for XCI in mammals. In this study, by using ChIRP-MS in a marsupial species and identifying 135 proteins that interact with RSX, of which 56 have orthologues in the mouse genome, the authors have found that Xist and RSX interactomes share the same protein-protein network with functions in post-transcriptional processing. Specifically, the authors identified ribonucleoproteins, such as HNRNPK, as common interacting proteins. By knocking-down HNRNPK in marsupial fibroblast cells, the authors observed a reduction of X chromosome silencing, which supports the functional significance of HNRNPK- RSX interaction for marsupial XCI. The ChIRP-MS experiments in marsupial cells and comparative analysis have been thoroughly performed, and the research findings provide valuable useful resources and new insights into the functional role of RSX in marsupial XCI and the evolution of sex chromosome dosage compensation in therian mammals.

Specific comments:

1. The authors pointed out that post-transcriptional regulation may be a common molecular machinery of XCI shared between eutherian and marsupial mammals. However, no specific proteins or factors have been identified or proposed as being involved in post-transcriptional control and XCI. The focus was instead placed on HNRNPK, which interacts with PRC1 in eutherian XCI. It would be helpful if the authors could clarify.

2. By using RNAi, the authors performed gene knockdown in marsupial. The knockdown efficiency for HNRNPK (by 24-35%) or for CKAP4 (by 53-55%) was not very high; given the observed effects on X chromosome were moderate (MSN bi-allelic frequency changed from 18% to 39%), it would be helpful if the authors could explain possible challenges for gene knockdown in marsupial cells. The knockdown efficiencies detected by RNA might differ from the protein level changes, which may have offset the functional consequences?

3. The authors validated the role of CKAP4 in marsupial XCI through RNAi. In marsupials, CKAP4 interacts with RSX, but in mice, there is no CKAP4-Xist interaction. Does marsupial CKAP4 protein differ significantly from mouse CKAP4 protein?

**Reviewer 2**

The study by McIntyre et al focuses on identifying the proteins interacting with RSX in Marsupial and compare the discovered interactome with the one from Xist which convey a similar function of X inactivation in Eutherians.

The question investigated by the authors is extremely interesting, and should be of general interest to the readership of Genome Biology. The presented investigation into the mechanisms of X inactivation in marsupial and the comparison to the well characterise mechanism in eutherian is very much exciting as those fundamental processes are being conveyed by two different key players: RSX and Xist.

The authors apply chromatin ChIRP-MS to identify the proteins that interact with RSX and compare those to the ones reported for Xist. Although the experiments are sounds, my major criticisms are with respect to the level of detail provided in the computational analyses performed currently preventing the analyses to be reproduced.

My apologies if I have missed this but there is no mention in the text or supplementary file regarding data availability, nor code availability for the analyses performed.

Gene set enrichment analysis: there is no description on how this was performed and more specifically on how the background set for the enrichment was selected, especially for assessing the overlap between the two GSEA, in this later part this should have been done only using 1:1 orthologues to limit potential biases.

Page 6: interactome analyses : "This was significantly higher than the 3,599 edges expected for a random set of 576 proteins (p < 1 x 10-16)." How the random set was selected? I guess that the authors selected truly random proteins, however to truly assess the hypothesis tested, the authors should have sampled 576 random proteins sharing the same gene functions as those found in the Xist and RSX interactome. It is very likely because of the functions (mRNA binding, spliceosomal complex) represented in those interactomes that the number of edges is already greater than a genome-wide background.

Page 9 and page 10: IDR enrichment analyses. Some functional categories are known to be enriched in IDR, therefore it is important that the authors ensure that their randomly selected proteins reflect the functional composition of their foreground data, especially due functional enrichment previously reported in the GSEA.

Minor comments
Page 6: "However, the other 56 had orthologues that were identified in the Xist interactome (497 proteins), and 33 RSX-interactors had orthologues that were identified in at least two of the Xist interactome studies." When defining orthologues are the authors focusing on 1:1 orthologues only? Additionally this sentence is a bit confusing, do the authors mean "of which 33 RSX-interactors ..."

Page 8: "CKAP4 which, unlike HNRNPK, has not been identified as an Xist interactor, and has no predicted interactions with any protein in either the Xist or RSX interactomes (Additional file 4: Dataset 3). In our RSX interactome, CKAP4 was the protein with the highest 20 fold-change (log2) enrichment relative to each of the controls in the native (uncrosslinked) ChIRPMS (Additional file 2: Dataset 2). " The authors should rephrase this section as the sentences do appear contradictory.

**Reviewer 3**

In this manuscript entitled "Functional coherence of the Xist and Rsx interactomes: X chromosome inactivation in marsupials", McIntyre & Waters and colleagues unveiled the interactome of Rsx, the master regulator of X-chromosome inactivation (XCI) in marsupials. The authors performed chromatin isolation by RNA purification followed by mass spectrometry (ChIRP-MS) in M. domestica and discovered 135 interactors of Rsx. The authors validated some of the identified factors and also tested the putative interaction between Rsx and a subset of candidate proteins not identified by ChIRP-MS but suspected to play a role in marsupial XCI. The authors unveiled that the interactomes of Rsx and Xist (major player of XCI in mouse) are largely overlapping with 56 orthologous proteins being identified in both systems and gene set enrichment analysis (GSEA) showing strong functional similarities. One class of proteins that was enriched in both Rsx and Xist interactome is the one of proteins with intrinsically disorder regions (IDRs), suggesting a role for these factors in the formation of RNA-proteins complexes that would favour XCI maintenance. Finally, the functional role of two identified Rsx interacting factors, HNRNPK and CKAP4, was tested by RNA FISH following RNAi in fibroblasts derived from M. domestica, and confirmed their contribution to XCI maintenance in marsupials.
This manuscript provides novel and exciting insights into the mechanisms underlying the fundamental function of Rsx, which is to achieve X-linked gene dosage compensation between the sexes. The described data is certainly timely and likely to be of great interest for the research community beyond the dosage compensation field. However in my opinion the main figures can be much improved and I do have a list of specific concerns and recommendations that I hope will contribute to ameliorate the m/s while increasing its already broad impact. I summarise them below.

1) The authors revealed extensive overlap between the Rsx and Xist interactomes but did not identify SPEN in the interactome of Rsx. This aspect is omitted in the m/s and in my opinion it should be addressed (or at least discussed). In fact, SPEN was identified as one of Xist's interactors in all Xist RNA pull-down studies (Chu et al. 2015; Minajigi et al. 2015, McHugh et al. 2015) and was proven to play a key role in Xist-mediated silencing during mouse embryonic development (Dossin et al. 2020). As the authors tested the interaction between Rsx and other candidate factors not identified by ChIRP-MS by RIP-qPCR (i.e. EZH2, HDAC2), I would recommend them to include SPEN in this set of experiments. Another way to consider this aspect could be to address whether any of the factors identified as part of the Rsx interactome are functional analogs of SPEN. For example, I wonder whether the authors identified proteins with the SPOC domain - which was proven to be key to mediate SPEN's role in XCI establishment.

2) In Fig 1B I would recommend the authors to add details about the identified functional enrichment terms for each cluster (as it's done in S1) and to label at least the main factors they discuss and/or validate.

3) In Fig 1C it is not clear why the authors decided to test proteins that were not identified by ChIRP-MS. How were these factors selected? Also, the data showing successful validation of HNRNPK is mentioned in the main text and figure legend but not shown in Fig 1C. The authors should include it for clarity.

4) I find the functional analysis of HNRNPK and CKAP4 very interesting. To make Fig 2 more impactful I would recommend the authors to add the FISH quantification to the main figure as a bar plot and to include data related to CKAP4 as well. I would also suggest moving Fig 2B to a separate figure.

5) In my opinion the authors should clarify what they conclude at page 9: "These data provide evidence that both HNRNPK and CKAP4 play roles in transcriptional silencing of the inactive X chromosome in marsupials". Given that the functional experiments were performed in fibroblast carrying a fully inactivated X chromosome, the observed increase in biallelic expression following knockdown of HNRNPK and CKAP4 provide evidence for the role of these factors in XCI maintenance rather than in the initiation of transcriptional silencing.

6) The authors show functional data to validate the role of CKAP4 in Rsx-mediated XCI, however they provide little details about the pathway and function in which this protein plays its role. Is there any obvious link to the known regulators of XCI? If not, is the validation of this factor providing the basis to explore novel mechanisms underlying XCI regulation in marsupials?

7) As I suggested above, I would include a separate figure to describe the observations related to the enrichment of proteins with IDRs in the Rsx interactome. Furthermore, given that Xist RNA forms complexes with the IDRs of its interacting proteins and having determined the high IDR scores of Rsx interactome, the addition of functional data to test the role of PTBP1 (or FUS, also identified in both Rsx and Xist interactomes) would certainly strengthen the authors' conclusions.

**Authors’ response**

Reviewer 1

Specific comment 1:

The authors pointed out that post-transcriptional regulation may be a common molecular machinery of XCI shared between eutherian and marsupial mammals. However, no specific proteins or factors have been identified or proposed as being involved in post-transcriptional control and XCI. The focus was instead placed on HNRNPK, which interacts with PRC1 in eutherian XCI. It would be helpful if the authors could clarify.

Response:

The list of interactome proteins that fall under each ontology term enriched in the *Xist* and *RSX* interactomes, were listed in Additional file 3 (tab 5). These terms include “post-transcriptional regulation of gene expression”, a category we highlighted due to its relevance. The *Xist* interactome features 74 genes associated with this term, and in the *RSX* interactome includes 19. Given their abundance, a comprehensive listing within the text was impractical.

For clarity, we have revised the description of the Additional files (page 30, lines 5 – 13) and made corresponding adjustments in the Results section with the following amendment:

Page 7, lines 9-10: “with Column E in each case listing the interactome proteins underlying each enriched ontology term”

The whole *Xist* and *RSX* interactomes are of interest. We had justified why HNRNPK was focused on, due to its presence in both the *RSX* and *Xist* interactomes. However, we now better justify in text why we focus on CKAP4 with the following amendment:

Page 9, line7-9: “In the *RSX* interactome, CKAP4 was unexpectedly the protein with the highest fold-change (20x) enrichment relative to controls in the native (uncrosslinked) ChIRP-MS (Additional file 2: Tab 2). Therefore, we used RNAi…”

Specific comment 2:

By using RNAi, the authors performed gene knockdown in marsupial. The knockdown efficiency for HNRNPK (by 24-35%) or for CKAP4 (by 53-55%) was not very high; given the observed effects on X chromosome were moderate (MSN bi-allelic frequency changed from 18% to 39%), it would be helpful if the authors could explain possible challenges for gene knockdown in marsupial cells. The knockdown efficiencies detected by RNA might differ from the protein level changes, which may have offset the functional consequences?

Response:

We have included reference to some hurdles faced when applying RNAi technology in non-eutherian models, and of the possible divergence between the knockdown levels of mRNA and of the corresponding protein. Amendments to the text are as follows:

Page 8, lines 11-12: using RNA interference (RNAi), “adapting eutherian-based construct design and delivery for our non-traditional model organism.”

Page 8, lines 17-19: “Knockdown efficiency was assayed by measuring transcript abundance using RT-qPCR. The knockdown effect on protein abundance may differ due to variations in post-transcriptional processing.”

Page 18-19): “The shRNA-expressing constructs were designed using a combination of online tools (BioSettia shRNA designer: http://biosettia.com/support/shrna-designer/, Invitrogen RNAi Designer: https://rnaidesigner.thermofisher.com/rnaiexpress/, InvivoGen siRNA Wizard v3.1: http://www.invivogen.com/sirnawizard/index.php, IDT RNAi Design Tool: <https://sg.idtdna.com/site/order/designtool/index/DSIRNA_CUSTOM>), and nucleotide BLAST of candidate sequences against the MonDom5 genome assembly (Ensembl release 84). For each target mRNA, candidate constructs were trialled for different regions of the mRNA to accommodate the possibility that binding may be impeded at certain sites by secondary structure or sequence variants.”

Specific comment 3:

The authors validated the role of CKAP4 in marsupial XCI through RNAi. In marsupials, CKAP4 interacts with RSX, but in mice, there is no CKAP4-Xist interaction. Does marsupial CKAP4 protein differ significantly from mouse CKAP4 protein?

Response:

Prompted by this insightful inquiry, we undertook comparative analysis of the CKAP4 protein sequence across broad range of species, including eutherians (mouse, human and hyrax - an afrotherian), monotremes (platypus and echidna) and eight marsupial species. The findings from this analysis are now presented in a new Figure 3. This analysis revealed a notable expansion of a glutamine (Q)-rich repeat at the N terminus of the monodelphis CKAP4 protein. To further explore this distinctive feature, we also employed AlphaFold to predict the tertiary structures of CKAP4 in monodelphis alongside three eutherian species (human, mouse and hyrax), now depicted in Figure 3B-C. These predications suggest a helical structure in the monodelphis CKAP N-terminus.

Poly Q tracts are well-documented for their propensity to form stable, interactions with RNA and proteins. The CAG-expanded RNA toxicity is a feature of interest in the context of neurodegenerative disorders, however Poly Q motifs also play a role in modulation of protein-protein interactions that produce aggregates. The expansion of the Poly Q tract in monodelphis N-terminus of CKAP4, particularly the propensity for adopting helical structures, suggests a potential mechanism by which it might exhibit altered functional interactions when compared to its eutherian counterparts. The potential for monodelphis CKAP4 to have enhanced affinity for RNA and/or protein could facilitate its interaction with *RSX*. This adaptation provides a plausible explanation for the distinctive role of monodelpthis CKAP4 in X chromosome inactivation.

These outcomes and implications have now been described in the results (Page 9 - 10), and the implications of the Poly Q expansion in marsupial CKAP4 have been discussed (Page 11, line 18 onwards). The sequence alignment and predicted structure models are incorporated into a new Figure 3.

Reviewer 2

General comment 1:

The authors apply chromatin ChIRP-MS to identify the proteins that interact with RSX and compare those to the ones reported for Xist. Although the experiments are sounds, my major criticisms are with respect to the level of detail provided in the computational analyses performed currently preventing the analyses to be reproduced.

My apologies if I have missed this but there is no mention in the text or supplementary file regarding data availability, nor code availability for the analyses performed.

Response:

MaxQuant protein group files are now provided as additional files 6-8. We have made code available in additional file 9.

General comment 2:

Gene set enrichment analysis: there is no description on how this was performed and more specifically on how the background set for the enrichment was selected, especially for assessing the overlap between the two GSEA, in this later part this should have been done only using 1:1 orthologues to limit potential biases.

Response:

We described the GSEA in the methods on page 23, lines 14-17. There are 108 1:1 orthologues between the *RSX* interactome and the human proteome. The remaining 25 are reciprocal best hits. Human orthologues for 2 RSX interactors could not be identified, and are not represented in the GSEA. This now better clarified in the manuscript:

Page 23, lines 3-9: “Human orthologues of *M. domestica* genes were identified using Ensembl 97 BioMart (http://jul2019.archive.ensembl.org/index.html). For genes without a one-to-one orthologue, a human orthologue or equivalent was identified using reciprocal protein BLASTs (https://www.uniprot.org/blast). There were two exceptions to this, where the MonDom5 gene did not have a human 1 to 1 orthologue (ENSMODG00000025105), or where the best reciprocal blast hit was already represented in the *RSX* interactome (ENSMODG00000013903). In these cases, the gene was excluded from downstream analysis.”

Specific comment 1:

Page 6: interactome analyses: "This was significantly higher than the 3,599 edges expected for a random set of 576 proteins (p < 1 x 10-16)." How the random set was selected? I guess that the authors selected truly random proteins, however to truly assess the hypothesis tested, the authors should have sampled 576 random proteins sharing the same gene functions as those found in the Xist and RSX interactome. It is very likely because of the functions (mRNA binding, spliceosomal complex) represented in those interactomes that the number of edges is already greater than a genome-wide background.

Response:

This is a STRING protein-protein interaction database prediction (string-db.org) (Szklarczyk, D, et al 2023). When provided with a list of proteins and species identification, STRING reports the number of edges between nodes, then also the expected number of edges when randomly sampling the same number of proteins from the proteome. The STRING analysis does not take into account function, which is subsequently determined with the GSEA on the whole interactome, and the clusters within the interactome. We have made this clear in the manuscript (page 6 - 7) with the following text:

“This was significantly higher than the 3,633 edges expected for a random set of 578 proteins selected from the same proteome (p < 1 x 10^-16^)”

Specific comment 2.

Page 9 and page 10: IDR enrichment analyses. Some functional categories are known to be enriched in IDR, therefore it is important that the authors ensure that their randomly selected proteins reflect the functional composition of their foreground data, especially due functional enrichment previously reported in the GSEA.

Response:

We now calculate XIST/RSX IDR scores v background IDR using background sets (for each of mouse and monodelphis) comprising the proteins corresponding to the gene sets supporting all ontology terms that are enriched (p < 0.001) in one or more of clusters 1, 2 and 3, as listed in additional file 3, column A of tabs 1, 2 & 3. Although p-values change a little, they all remain significant for the comparisons we made. This is described in the text as follows:

Page 10 - 11: “The background proteome subset comprised all proteins within the ontology terms (additional file 3, tabs 1-3, column A) enriched (p < 1 x 10^-3^) in one or more of the combined interactome clusters 1, 2 and 3.”

Page 24, lines 6-17: “The background proteome subset comprised all proteins within the ontology terms (additional file 3, tabs 1-3, column A) enriched (p < 1 x 10^-3^) in one or more of the combined interactome clusters 1, 2 and 3. Gene sets were obtained for each species from the GMT files for each of GO:BP, GO:MF, GO:CC (<https://biit.cs.ut.ee/gprofiler/gost>, accessed 24 February 2024). Mouse orthologues of proteins in the *RSX* interactome were identified using Ensembl 97 BioMart (<http://jul2019.archive.ensembl.org/index.html>).

For genes without an identified one-to-one orthologue, a mouse orthologue was identified using reciprocal best hit protein BLAST (<https://www.uniprot.org/blast>). Three *RSX* interactors were not represented in the mouse orthologue set, either because no 1 to 1 orthologue was identified (ENSMODG00000006291, ENSMODG00000024476), or where the best reciprocal BLAST hit was already in the *RSX* interactome (ENSMODG00000008362).”

Minor comment 1.

Page 6: "However, the other 56 had orthologues that were identified in the Xist interactome (497 proteins), and 33 RSX-interactors had orthologues that were identified in at least two of the Xist interactome studies." When defining orthologues are the authors focusing on 1:1 orthologues only? Additionally this sentence is a bit confusing, do the authors mean "of which 33 RSX-interactors ..."

Response:

We are focusing on 1 to 1 orthologues or reciprocal best hits. This is now fully described in the Methods (page 23, lines 3-9). We have also reworded the sentence for clarity:

Page 6, lines 5-9: “These proteins were included in the *RSX* interactome, bringing the total to 135 proteins. We considered whether proteins of the *RSX* interactome had orthologues in the *Xist* interactome. Of the 135 *RSX*-interactors, 81 did not have orthologues in the *Xist* interactome, so were specific to the *RSX* interactome. The remaining 54 *RSX*-interactors had orthologues that were identified in the *Xist* interactome (which comprises 497 proteins in total).”

Minor comment 2.

Page 8: "CKAP4 which, unlike HNRNPK, has not been identified as an Xist interactor, and has no predicted interactions with any protein in either the Xist or RSX interactomes (Additional file 4: Dataset 3). In our RSX interactome, CKAP4 was the protein with the highest 20 fold-change (log2) enrichment relative to each of the controls in the native (uncrosslinked) ChIRPMS (Additional file 2: Dataset 2)." The authors should rephrase this section as the sentences do appear contradictory.

Response:

For clarity we have rephrased these sentences to read:

Page 9, lines 6-9: “CKAP4 has not been identified as an *Xist* interactor, and has no predicted interactions with any protein in either the *Xist* or *RSX* interactomes (Additional file 4: Tab 3). In the *RSX* interactome, CKAP4 was unexpectedly the protein with the highest fold-change (20x) enrichment relative to controls in the native (uncrosslinked) ChIRP-MS (Additional file 2: Tab 2).”

Reviewer 3

Comment 1.

1) The authors revealed extensive overlap between the Rsx and Xist interactomes but did not identify SPEN in the interactome of Rsx. This aspect is omitted in the m/s and in my opinion it should be addressed (or at least discussed). In fact, SPEN was identified as one of Xist's interactors in all Xist RNA pull-down studies (Chu et al. 2015; Minajigi et al. 2015, McHugh et al. 2015) and was proven to play a key role in Xist-mediated silencing during mouse embryonic development (Dossin et al. 2020). As the authors tested the interaction between Rsx and other candidate factors not identified by ChIRP-MS by RIP-qPCR (i.e. EZH2, HDAC2), I would recommend them to include SPEN in this set of experiments. Another way to consider this aspect could be to address whether any of the factors identified as part of the Rsx interactome are functional analogs of SPEN. For example, I wonder whether the authors identified proteins with the SPOC domain - which was proven to be key to mediate SPEN's role in XCI establishment.

Response:

Given that SPEN was reported to be required for *Xist* upregulation during initiation of XCI (Robert-Finestra, T., et al 2021), we were not necessarily expecting to detect SPEN as the fibroblast model we look at is with respect to maintenance of XCI, not initiation. However, we acknowledge the importance of SPEN and is now discuss.

Page 13, lines 1-6: “Cluster 3 contained proteins typically associated with XCI, including those involved in epigenetic regulation of transcriptional silencing, histone modifications and heterochromatin. These proteins include SPEN, which was identified in all of the *Xist* ChiRP-MS studies but was absent from the *RSX* interactome. SPEN is required for upregulation of *Xist* during XCI initiation (41), but becomes less important during the maintenance stage (42). Therefore, it was not unexpected that SPEN was absent from the RSX interactome in our fibroblast model, which represents XCI maintenance.”

Comment 2.

2) In Fig 1B I would recommend the authors to add details about the identified functional enrichment terms for each cluster (as it's done in S1) and to label at least the main factors they discuss and/or validate.

Response:

We attempted drafting a figure with the main enriched terms in Fig 1B (as in S1), however the panel became too busy. The main terms the reviewer refers to are already in Fig 1D. To make a better connect between Fig 1B and Fig 1D, we have switched Fig 1D into the Fig 1C position and made the appropriate changes to the figure legend.

Comment 3.

3) In Fig 1C it is not clear why the authors decided to test proteins that were not identified by ChIRP-MS. How were these factors selected? Also, the data showing successful validation of HNRNPK is mentioned in the main text and figure legend but not shown in Fig 1C. The authors should include it for clarity.

Response:

The ChIRP-MS and RIP-qPCR experiments were conducted in parallel. The selection of proteins for RIP-qPCR were based on their known (or potential) roles in chromatin modification and XCI. We have made this clear in the manuscript with the following:

Page 5, lines 15-20: “The RIP targets were chosen for their potential role in XCI. In eutherian models SUZ12/EZH2 (core components of PRC2), HDAC2, HNRNPK and CTCF have roles in eutherian XCI. MBD2+3, MBD4, and MECP2 bind methylated DNA, and are involved in chromatin remodelling and gene regulation. The histone marks H3K9me3 and H3K27me3 are known to accumulate on the inactive X in marsupials. PCAF is an acetyl transferase, so served as a negative control (Figure 1D).”

The HNRNPK RIP-qPCR result was not included in Fig 1C because it was previously published. This citation was included in the manuscript main text, and has now also been included in the figure ledged. We are unsure if the result can be presented again here.

Comment 4.

4) I find the functional analysis of HNRNPK and CKAP4 very interesting. To make Fig 2 more impactful I would recommend the authors to add the FISH quantification to the main figure as a bar plot and to include data related to CKAP4 as well. I would also suggest moving Fig 2B to a separate figure.

Response:

We have moved the FISH quantification data from Additional file 6 to the Figure 2 legend, and also included representative images of the CKAP4 knockdown RNA FISH. As recommended by the reviews Figure 2B has been moved to a new Figure 3, which includes further examination of the structure of CKAP4. Please see response to reviewer 1 specific comment 3.

Comment 5.

5) In my opinion the authors should clarify what they conclude at page 9: "These data provide evidence that both HNRNPK and CKAP4 play roles in transcriptional silencing of the inactive X chromosome in marsupials". Given that the functional experiments were performed in fibroblast carrying a fully inactivated X chromosome, the observed increase in biallelic expression following knockdown of HNRNPK and CKAP4 provide evidence for the role of these factors in XCI maintenance rather than in the initiation of transcriptional silencing.

Response:

For clarity about maintenance of XCI (rather than initiation) we have reworded to the following:

Page 7 lines 1-3: “This outcome was observed across two independent experiments, and provides evidence that HNRNPK plays a role in maintenance of transcriptional silencing on the inactive X chromosome in marsupials.”

Page 9 lines 12-13: “This suggests that CKAP4 plays a role in maintenance of monodelphis XCI.”

Comment 6.

6) The authors show functional data to validate the role of CKAP4 in Rsx-mediated XCI, however they provide little details about the pathway and function in which this protein plays its role. Is there any obvious link to the known regulators of XCI? If not, is the validation of this factor providing the basis to explore novel mechanisms underlying XCI regulation in marsupials?

Response:

Please see response to reviewer 1 specific comment 3. These comments led us to further investigate CKAP4.

Comment 7.

7) As I suggested above, I would include a separate figure to describe the observations related to the enrichment of proteins with IDRs in the Rsx interactome. Furthermore, given that Xist RNA forms complexes with the IDRs of its interacting proteins and having determined the high IDR scores of Rsx interactome, the addition of functional data to test the role of PTBP1 (or FUS, also identified in both Rsx and Xist interactomes) would certainly strengthen the authors' conclusions.

Response:

We initially chose HNRNPK because of its central role in *XIST* silencing. We then chose to examine CKAP4 because it was our top hit in the *RSX* interactome, not present in any of the *Xist* interactomes, and completely unexpected. It would be ideal to examine all the important proteins in the *RSX* interactome, however, due to time constraints, we are unable to conduct further knockdowns of other *RSX* interactors. We believe that the inclusion of CKAP4 comparative analysis enhances the manuscript.

**2^nd^ round**

**Reviewer 1**

The authors have addressed all my previous comments. The new Figure 3 and the comparative analysis of CKAP4 are informative and have improved the manuscript.

**Reviewer 2**

I am grateful to the authors for addressing my comments

**Reviewer 3**

I would like to thank the authors for their response to my comments and recommendations. I find the revised version of the m/s much improved. My suggestions were either implemented in a very satisfactory way or else, the authors explained the technical/time limitations. My feedback for the authors can therefore only be positive.
